# Risk and adverse clinical outcomes of thrombocytopenia among patients with solid tumors—a Danish population-based cohort study

**DOI:** 10.1038/s41416-024-02630-w

**Published:** 2024-03-06

**Authors:** Kasper Adelborg, Katalin Veres, Erzsébet Horváth-Puhó, Mary Clouser, Hossam Saad, Henrik Toft Sørensen

**Affiliations:** 1 Department of Clinical Epidemiology, Department of Clinical Medicine, Aarhus University and Aarhus University Hospital, Aarhus, Denmark; 2 Department of Clinical Biochemistry, Thrombosis and Hemostasis Research Unit, Aarhus University Hospital, Aarhus, Denmark; 3 Amgen Inc., Thousand Oaks, CA USA

**Keywords:** Tumour biomarkers, Risk factors

## Abstract

**Background:**

Knowledge about thrombocytopenia among patients with solid tumors is scarce. We examined the risk of thrombocytopenia among patients with solid tumors and its association with adverse outcomes.

**Methods:**

Using Danish health registries, we identified all patients with incident solid tumors from 2015-2018 (*n* = 52,380) and a platelet count measurement within 2 weeks prior to or on their cancer diagnosis date. The risk of thrombocytopenia was categorized as grades 0 (any platelet count × 10^9^/L): <150; 1: <100; 2: <75; 3: <50; 4: <25, and 5: <10. To study the outcomes, each patient with thrombocytopenia was matched with up to five cancer patients without thrombocytopenia by age, sex, cancer type, and stage. Cox regression was used to compute hazard ratios (HRs) of bleeding, transfusion, or death, adjusting for confounding factors.

**Results:**

The 1-year risk of thrombocytopenia was 23%, increasing to 30% at 4 years. This risk was higher in patients receiving chemotherapy (43% at 1 year and 49% at 4 years). Overall, patients with thrombocytopenia had higher 30-days rates of bleeding (HR = 1.72 [95% confidence interval, CI: 1.41–2.11]). Thrombocytopenia was also associated with an increased rate of transfusion, and death, but some of the risk estimates were imprecise.

**Conclusions:**

The risk of thrombocytopenia was substantial among patients with solid tumors and associated with adverse outcomes.

## Introduction

Thrombocytopenia occurs frequently among patients with cancer [1]. Contributing factors are multifactorial and include use of various drugs, bleeding, tumor involvement of the bone marrow, microangiopathic disorders, disseminated intravascular coagulation, and immune disorders [1, 2].

Despite the increasing number of cancer survivors [3], knowledge is scarce about risks and prognosis related to thrombocytopenia in the routine clinical care setting. The incidence of thrombocytopenia in a population of solid tumor patients treated with chemotherapy was 10–13% in two studies based on large United States electronic medical record and claims datasets [4, 5]. In another study of adult solid tumor patients receiving chemotherapy, the 3-month risk of thrombocytopenia was 13% [6]. Thrombocytopenia can have important clinical consequences, such as postponement of or reduction in cancer treatment dose. Moreover, some prior studies found that thrombocytopenia was associated with increased risk of bleeding [7]. Prior studies’ limitations include inability to track patients across transitions between sites of care and insurance coverage. Further, the studies used broad and inconsistent cutoffs for thrombocytopenia, making it infeasible to disentangle the associations between different severities of thrombocytopenia and bleeding risk [7]. Studies were also small in size, were published more than 10 years ago, or were based on diagnosis codes, which do not reflect the biological trajectories of platelet count measures [7]. Finally, prior evidence focused on thrombocytopenia among hematologic cancer patients [7], and it is questionable whether this knowledge also applies to solid tumor patients. Firmer evidence is therefore needed about the burden of thrombocytopenia among solid tumor patients within a uniform population-based setting.

This would help to guide follow-up programs and to understand the potential impact of platelet transfusion and emerging drug therapies for thrombocytopenia. Further, it provides the foundation for guidelines on prevention and treatment strategies for solid tumor patients. We examined the occurrence, risk factors, and adverse clinical outcomes of thrombocytopenia among solid tumor patients with incident cancer and among solid tumor patients starting chemotherapy.

## Methods

### Study design and settings

This nationwide population-based cohort study was conducted in Denmark, which has a government-funded healthcare system providing free access to healthcare for all residents [8, 9]. Central personal registry numbers were used to link data from the Danish Cancer Registry [10], the Danish Register of Laboratory Results for Research [11], the Danish National Patient Registry [12], and the Danish Civil Registration System [13]. These data sources are described in Supplementary Table 1. According to Danish legislation, approval from an ethics committee is not required for registry-based studies. The study was reported to the Danish Data Protection Agency (record no.: 1819).

### Study cohorts

The first study cohort consisted of all patients diagnosed with incident solid tumors between 2015 and 2018 who were ≥18 years and had a platelet count measurement within the 2 weeks prior to or on their cancer diagnosis date. The second cohort comprised patients who started chemotherapy at any time following their incident cancer diagnosis.

### Thrombocytopenia, anemia, neutropenia, and leukopenia

Clinical data were linked with routine laboratory test results in the primary care and hospital settings. Outcomes were defined according to the Common Terminology Criterial for Adverse Events by National Cancer Institute adapted for real-world data. Based on platelet count levels, thrombocytopenia was categorized as present/absent and by severity as grades 0 (any platelet count × 10^9^/L): <150; 1: <100 × 10^9^/L; 2: <75 × 10^9^/L; 3: <50 × 10^9^/L; 4: <25 × 10^9^/L, and 5: <10 × 10^9^/L. Isolated thrombocytopenia was considered, i.e., absence of anemia, neutropenia, or leukopenia one day prior to or on the date of platelet count measurement. Combinations of “penias” were defined as thrombocytopenia with anemia only, thrombocytopenia with anemia and neutropenia, and thrombocytopenia with neutropenia only. Each combination of conditions had to be present on the same date ±1 day.

To contextualize risk estimates for thrombocytopenia, we also examined the risk of:

- Anemia: Any and by severity (grade 1: Hemoglobin <lower normal limit; grade 2: <6.2-mmol/L; and grade 3: <4.9 mmol/L.
- Neutropenia: Any and by severity (grade 1: <lower normal limit; grade 2: <1.5 × 10^9^/L; grade 3: <1.0 × 10^9^/L; grade 4: <0.5 × 10^9^/L; grade 5: <0.5 × 10^9^/L and death within 1 week after observation.
- Leukopenia: Leukocytes <4 × 10^9^/L.

### Prognosis associated with thrombocytopenia

Each solid tumor patient with thrombocytopenia was matched with up to 5 solid tumor patients without thrombocytopenia according to age, sex, cancer type, cancer stage, and duration of cancer/duration of chemotherapy. Outcomes assessed during 30 days of follow-up were any bleeding leading to hospitalization; any transfusion, including platelet transfusion, red blood cell transfusion, or plasma transfusion; and all-cause mortality. Site-specific bleeding leading to hospitalization was also examined. This included spontaneous and traumatic intracranial bleeding, respiratory tract, gastrointestinal, urinary tract, hemorrhagic cystitis, or anemia from acute bleeding.

### Covariates

We obtained data on age group, sex, year of study entry, cancer stage, bone metastasis, liver metastasis, solid cancer type, and Charlson Comorbidity Index (CCI) score. Medical conditions were chronic liver disease, disseminated intravascular coagulation, immune thrombocytopenia, and hemolytic uremic syndrome/thrombotic uremic syndrome. All available primary and secondary hospital-based discharge diagnoses (except emergency room diagnoses) prior to the index date, but after 1977, were used.

### Statistical analyses

The index date was the date of incident cancer diagnosis or the date of chemotherapy initiation. All patients contributed risk time from their index date until emigration, occurrence of an outcome of interest, death, or end of the study period (31 December 2018), whichever came first. Patients could be included in both the cohort of all incident cancer patients and the cohort of patients who commenced chemotherapy. We tabulated patient characteristics for the covariates described previously. The prevalence of thrombocytopenia, anemia, neutropenia, and leukopenia was calculated using the most recent observation within 2 weeks prior to or on the index date. All analyses were performed separately for the cohort of incident solid tumor patients and for the cohort of solid tumor patients starting chemotherapy. All definitions used in the study are provided in Supplementary Tables 2–5. We tabulated the proportion of cancer patients with a platelet count measurement using different time windows to evaluate whether the patients were captured by the laboratory database. This was done by study period, Danish region, age group, and cancer type. To ensure compliance with the data protection regulations, we rounded all the presented patient numbers to the nearest five.

### Risk of thrombocytopenia

We graphically illustrated the cumulative risk of thrombocytopenia, accounting for the competing risk of death [14]. We also calculated 90-day, 1-year, and 4-year risk estimates. In the overall analysis, only patients with normal levels of the individual biomarkers on their index date were considered “at risk”. In analyses of severity according to grade, patients with low-grade levels at baseline could be “at risk” of developing more severe grades during follow-up. Thus, patients were “at risk” for different outcome categories and for a more severe grade within each outcome category. For example, a patient may have experienced a higher grade of thrombocytopenia before a lower grade was observed (e.g., grade 4 before grade 2). In the grade 2 analysis, we terminated follow-up on the date of the grade 4 observation, assuming that they experienced grade 2 thrombocytopenia just before grade 4.

Further, to evaluate the burden of thrombocytopenia among affected patients, we tabulated the median numbers of “episodes” during follow-up. As well, median duration was calculated as the number of days between the initial observation and occurrence of a measurement within the normal range, or death, emigration, or end of follow-up, whichever came first. In this analysis, patients could contribute more than one episode.

### Risk factors for thrombocytopenia

We assessed risk factors for thrombocytopenia according to baseline characteristics. Risk factors included age (reference group: 18–30 years), sex (reference group: male), cancer stage (reference group: localized), cancer type (reference group: breast cancer), CCI score (reference group: CCI score of 0), and cancer treatment within 30 days before and 7 days after the index date (reference group: non-receipt of a specific treatment). For this analysis, the index date was 7 days after the cancer diagnosis/chemotherapy date, to avoid immortal time bias. For solid tumor patients initiating chemotherapy, additional risk factors included type of chemotherapy (focusing on the 10 most commonly used drug regimens). 90-day, 1-year, and 4-year hazard ratios (HRs) with 95% confidence intervals (CIs) were computed using a multivariable Cox proportional hazards regression model. The HRs were both unadjusted and adjusted by age, sex, cancer stage, cancer type, CCI score, and cancer treatment.

### Adverse outcomes following thrombocytopenia

To address the prognostic impact of thrombocytopenia on adverse outcomes, we sampled five comparators without thrombocytopenia (with replacement) for each patient who experienced thrombocytopenia [15]. For the prognosis part of the study, patients with thrombocytopenia (platelet count level ×10^9^/L) were categorized into mutually exclusive groups: grade 0 = 100–149, grade 1 = 75–99, grade 2 = 50–74, grade 3 = 25–49, grade 4 = < 25-10, and grade 5 = < 10 and were followed for a maximum of 30 days. These analyses included only patients having at least 30 days between their index date and administrative study end (31 December, 2018). Incidence rates of study outcomes per 1000 person–years were calculated. Cox proportional hazards regression models were fitted to estimate HRs, adjusting for CCI score and matching factors, by design. In additional analyses, we examined associations between different combinations of thrombocytopenia, anemia, and neutropenia and all-cause mortality.

## Results

**Fig. 1:**
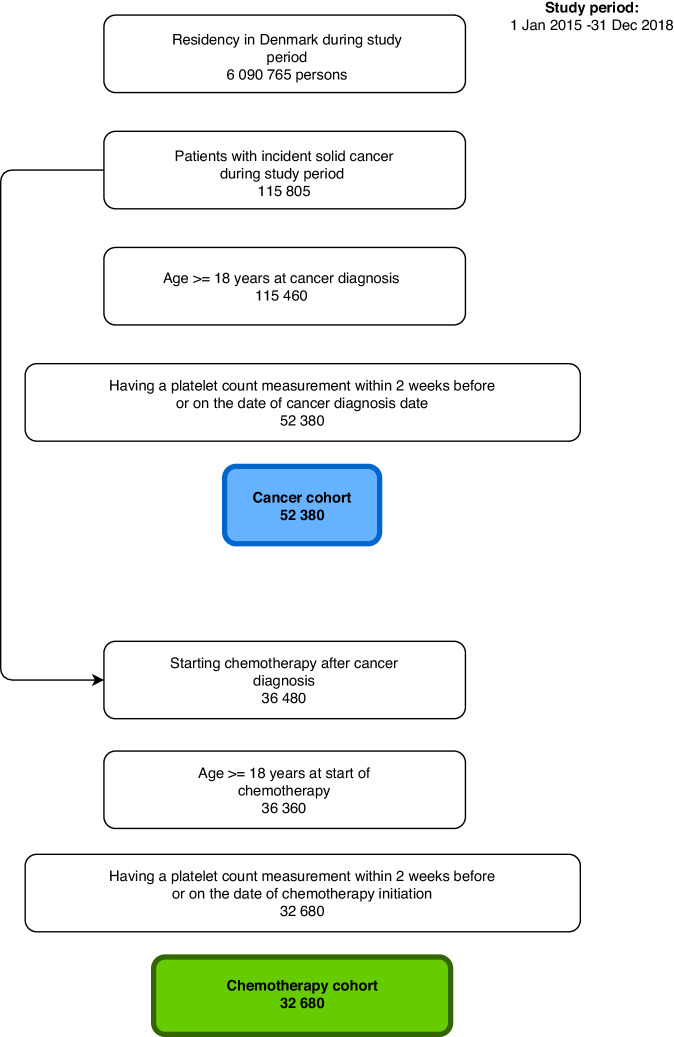
Flow chart of study inclusion. Two cohorts were identified: 1) An incident cancer cohort and 2) Incident cancer patients starting chemotherapy. For the cancer cohort, the proportion of patients with a platelet count measurement was 65%. This number varied by geographical region. Capital Region: 74%, Central Region: 71%, Northern Region: 67%, Zealand Region: 67%, Southern Region: 60%. For the cancer cohort, the distribution of cancer patients with a platelet count measurement was stable during the study period in the Capital Region, Central Region, Northern Region, and Zealand Region. In Southern Region, the proportion of cancer patients with a platelet count measurement was 22% in 2015, while it was 71% in 2016, 72% in 2017, and 72% in 2018. For the chemotherapy cohort, the proportion of patients with a platelet count measurement was 96%.

We identified 115,460 adult patients with incident solid tumors (Fig. 1). After restricting this population to patients with a platelet count measurement within 2 weeks prior to or on the index date, the cohort comprised 52,380 patients (median age = 70 years, interquartile range: 61-77 years). Changing the time windows for a platelet count measurement showed that around 95% of the patients had a measurement at any time points (Supplementary Table 6). Among patients with solid tumors, 32% had localized disease, 17% had regional spread, 27% had metastasis, and 25% had an unknown or missing cancer stage. The most common cancer types were gastrointestinal cancers (32%), respiratory cancers (23%), urogenital cancers (20%), and breast cancer (13%). The majority (56%) of patients had no comorbidity (evaluated by their CCI score) at study inclusion. Among 4%, thrombocytopenia of any grade was observed during the 2 weeks preceding study inclusion (grade 0: 3%, grade 1: 1%, and grades 2–4: 0%) (Table 1). On the cancer diagnosis date, anemia was present among 36% of solid tumor patients.

**Table 1:** Characteristics of solid tumor patients with incident cancer and solid tumor patients starting chemotherapy, 2015–2018, Denmark.

	Incident cancer cohort		Cancer patients starting chemotherapy	
	*N*	%	*N*	%
Overall	52,380	100	32,680	100
Age groups, years				
18–30	465	1	430	1
31–50	4130	8	4715	14
51–70	23,495	45	17,740	54
70+	24,290	46	9790	30
**Female**	26,395	50	18,095	55
Year of study entry				
2015–2016	24,855	47	15,025	46
2017–2018	27,525	53	17,655	54
Cancer stage				
Localized	16,525	32	7285	22
Regional spread	8695	17	8940	27
Distant metastasis	14,330	27	9495	29
Unknown	10,415	20	5265	16
Missing	2415	5	1695	5
Metastasis				
Liver	205	0	1225	4
Bone	80	0	490	1
Cancer type				
Mouth and pharynx	1035	2	975	3
Gastrointestinal	16,555	32	10,570	32
Respiratory	11,905	23	6660	20
Bone	85	0	45	0
Malignant melanoma	660	1	270	1
Soft tissue	805	2	565	2
Breast	6930	13	6615	20
Urogenital	10500	20	5315	16
Central nervous system	1550	3	925	3
Endocrine glands	400	1	140	0
Unspecified	1955	4	600	2
Charlson Comorbidity Index score				
0	29,170	56	21,305	65
1–2	17,685	34	9625	29
3+	5525	11	1745	5
Medical conditions				
Chronic liver disease	1560	3	615	2
Disseminated intravascular coagulation	10	0	5	0
Immune thrombocytopenia	60	0	35	0
Hemolytic uremic syndrome/thrombotic thrombocytopenic purpura	5	0	0	0
Laboratory data^a^				
Platelet count levels (×10^9^/L)				
Normal	50,125	96	31,975	98
Any thrombocytopenia ( < 150)	2260	4	705	2
Grade 0 (100-149)	1645	3	550	2
Grade 1 (75-99)	315	1	75	0
Grade 2 (50-74)	165	0	45	0
Grade 3 (25-49)	105	0	25	0
Grade 4 ( < 25-10)	20	0	10	0
Grade 5 ( < 10)	10	0	0	0
Anemia				
Normal hemoglobin	32,720	62	21,895	67
Any anemia (Hgb <7.3 mmol/l for women and 8.3 mmol/L for men)	19,020	36	10,755	33
Missing	640	1	30	0
Grade 1 (Hgb <lower limit of normal range-6.2 mmol/L)	13,670	26	9065	28
Grade 2 (Hgb <6.2-4.9 mmol/L)	4600	9	1580	5
Grade 3 (Hgb <4.9 mmol/L)	750	1	105	0
Neutropenia				
Normal neutrophiles	32,605	62	27,380	84
Any neutropenia (<2 ×10^9^/L)	340	1	530	2
Missing	19,435	37	4765	15
Grade 1 (2–1.5 × 10^9^/L)	230	0	410	1
Grade 2 (<1.5–1.0 × 10^9^/L)	70	0	85	0
Grade 3 (<1.0–0.5 ×10^9^/L	30	0	25	0
Grade 4 (<0.5 × 10^9^/L)	10	0	5	0
Grade 5: (<0.5 × 10^9^/L and death within 1 week after observation)	0	0	.	.
Leukopenia				
Normal leukocytes	46,590	89	32,020	98
Any leukopenia (<4×10^9^/L)	675	1	570	2
Missing	5115	10	85	0
Cancer treatment within 90 days before and after index date				
Radiation therapy	10,580	20	7745	24
Immunotherapy	4480	9	6200	19
Chemotherapy	17,820	34	32,680	100

To ensure compliance with the data protection regulations, we rounded all the presented patient numbers to the nearest five.

^a^The most recent observation at the index date or within 6 months before.

### Risk of thrombocytopenia

**Fig. 2:**
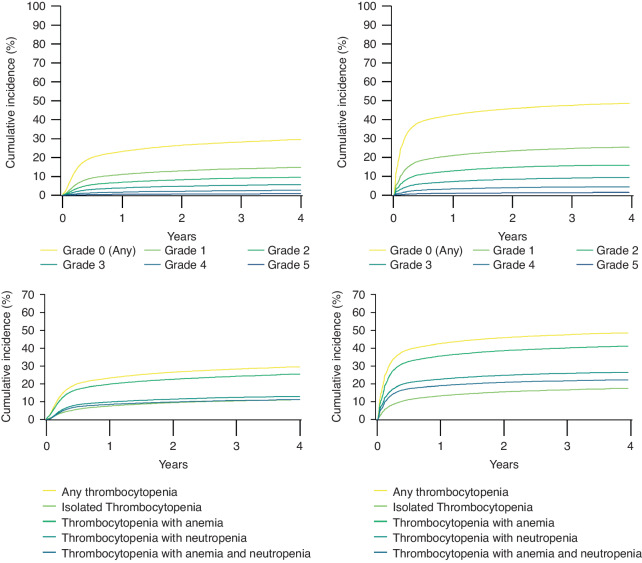
Cumulative incidence risk of thrombocytopenia for solid tumor patients with incident cancer and for solid tumor patients initiating chemotherapy.

The 90-day, 1-year, and 4-year risks of new-onset thrombocytopenia were 14% (95% CI: 14–15%), 23% (95% CI: 23–24%), and 30% (95% CI: 29–30%), respectively (Fig. 2 and Supplementary Table 7). Among those who initiated chemotherapy (n = 32,680), the 90-day, 1-year, and 4-year risks of thrombocytopenia were 34% (95% CI: 33–34%), 43% (95% CI: 42–43%), and 49% (95% CI: 48–49%), respectively. (Fig. 2 and Supplementary Table 7). Grades 0–2 thrombocytopenia occurred more frequently than grades 3-4. The number and duration of episodes of thrombocytopenia are shown in Supplementary Table 8 (median episodes = 1, interquartile range: 1–3 episodes and median duration = 16 days, interquartile range: 8–41 days).

### Risk of anemia, neutropenia, and leukopenia

Among solid tumor patients with incident cancer, the 90-day, 1-year, and 4-year risks of new-onset anemia were 52% (95% CI: 51–52%), 64% (95% CI: 64–65%), and 72% (95% CI: 72–73%), respectively (Fig. 1 and Supplementary Table 8). Neutropenia and leukopenia occurred less frequently. The number and duration of episodes of anemia, neutropenia, and leukopenia are shown in Supplementary Table 8 and the risk estimates in cancer patients initiating chemotherapy are presented in Supplementary Fig. 1.

### Risk factors for thrombocytopenia

Rates of thrombocytopenia were increased in patients with a high burden of comorbidity, in men, and in patients with an advanced cancer (Supplementary Tables 9 and 10). The risk of thrombocytopenia was also more pronounced in some types of cancer (e.g., bone, gastrointestinal, and respiratory cancers compared with breast cancer), and in the 90 days following radiation therapy. Advanced age and immunotherapy were not associated with increased risk of thrombocytopenia. The effect estimates of individual chemotherapy regimens for the risk of thrombocytopenia were generally imprecise, but some heterogeneity was observed.

### Adverse outcomes following thrombocytopenia

Solid tumor patients with thrombocytopenia had higher rates of any bleeding leading to hospitalization than patients without thrombocytopenia (overall adjusted HR = 1.67 [95% CI: 1.36–2.04], grade 0: adjusted HR = 1.54 [95% CI: 1.22–1.95], grade 1: adjusted HR = 2.03 [95% CI: 1.18–3.49], and grade 2: adjusted HR = 1.58 [95% CI: 0.72–3.45]). Grades 3-4 thrombocytopenia also showed an increased rate of hospitalization for bleeding, but estimates were imprecise (Table 2). For grade 5, we detected too few outcomes to estimate the hazard ratios. The overall estimate was similar in the population of solid tumor patients starting chemotherapy (adjusted HR = 1.48 [95% CI: 1.09–2.00]). Site-specific bleeding results were generally imprecise (Supplementary Tables 11 and 12).

**Table 2:** Adverse clinical outcomes associated with thrombocytopenia among patients with incident solid tumors.

	Incidence rate per 1000 person–years (95% confidence interval)		Hazard ratio (95% confidence interval)	
Adverse outcomes by grade	Patients without thrombocytopenia	Patients with thrombocytopenia	Unadjusted	Adjusted
Bleeding leading to hospitalization				
Any	76.39 (69.04–83.74)	567.60 (471.13–664.06)	1.71 (1.40–2.08)	1.67 (1.36–2.04)
Grade 0	73.49 (65.51–81.46)	507.60 (405.52–609.67)	1.57 (1.24–1.98)	1.54 (1.22–1.95)
Grade 1	92.07 (66.55–117.59)	759.04 (426.38–1091.7)	2.12 (1.25–3.59)	2.03 (1.18–3.49)
Grade 2	91.97 (55.17–128.77)	798.71 (303.66–1293.8)	1.97 (0.94–4.12)	1.58 (0.72–3.45)
Grade 3	92.39 (42.17–142.61)	450.93 (0.00–961.20)	1.34 (0.38–4.75)	1.52 (0.41–5.67)
Grade 4	50.41 (0.00–120.28)	2479.1 (0.00–5284.4)	7.18 (1.20–43.03)	10.23 (1.04–100.61)
Grade 5	-	4623.4 (0.00–11031)	-	-
Any transfusion				
Any	6.79 (4.60–8.97)	204.38 (147.15–261.61)	6.67 (4.29–10.35)	6.96 (4.41–10.99)
Grade 0	6.06 (3.78– 8.35)	125.31 (75.18–175.45)	4.30 (2.44–7.59)	4.42 (2.48–7.87)
Grade 1	5.50 (0.00–11.73)	220.56 (44.08–397.05)	9.53 (2.38–38.15)	11.87 (2.13–66.15)
Grade 2	22.91 (4.58–41.25)	393.67 (48.60–738.74)	3.74 (1.14–12.30)	4.24 (1.08–16.61)
Grade 3	7.07 (0.00– 20.92)	1190.2 (365.44–2015.0)	-	-
Grade 4	-	4542.9 (560.87–8524.9)	-	-
Grade 5	-	2006.9 (0.00–5940.3)	-	-
Death				
Any	780.48 (757.04–803.93)	3780.4 (3534.9–4025.9)	3.66 (3.35–4.00)	3.54 (3.24–3.87)
Grade 0	746.53 (721.15–771.91)	3249.9 (2994.9–3504.9)	3.15 (2.84–3.49)	3.06 (2.76–3.39)
Grade 1	938.85 (857.53–1020.2)	4690.6 (3881.2–5500.1)	4.21 (3.31–5.35)	4.07 (3.19–5.20)
Grade 2	869.89 (756.97–982.80)	6149.8 (4793.6–7505.9)	6.33 (4.50–8.92)	6.19 (4.38–8.73)
Grade 3	989.42 (825.52–1153.3)	8406.5 (6243.0– 10570)	8.47 (5.46–13.14)	8.58 (5.47–13.44)
Grade 4	905.58 (609.76–1201.4)	13045 (6652.8– 19437)	12.67 (4.95–32.43)	13.56 (5.15–35.73)
Grade 5	1367.0 (697.18–2036.9)	9979.5 (1232.1– 18727)	-	-

The reference group is a matched comparison cohort without thrombocytopenia. The unadjusted analyses are controlled by matching factors by design. The analyses are adjusted for Charlson Comorbidity Index scores and by matching factors by design.

All grades of thrombocytopenia were associated with an increased rate of transfusion (overall for incident solid tumors: adjusted HR = 6.96 [95% CI: 4.41–10.99]) and with death (adjusted HR = 3.54 [95% CI: 3.24–3.87]) (Tables 2 and 3). The association with death generally increased in magnitude with decreasing platelet count levels. The adjusted HRs of death according to combinations of thrombocytopenia, anemia, and neutropenia are shown in Supplementary Table 13. The HRs were higher for thrombocytopenia in combination with other penias than for isolated thrombocytopenia. However, even patients with low-grade isolated thrombocytopenia had a higher mortality rate than patients without thrombocytopenia. Using 1:1 matching led to more imprecise estimates, but the results were broadly unchanged (Supplementary Tables 14-15).

**Table 3:** Adverse clinical outcomes associated with thrombocytopenia among patients with incident solid tumors who were initiating chemotherapy.

	Incidence rate per 1000 person-years (95% confidence interval)		Hazard ratio (95% confidence interval)	
Adverse outcomes by grade	Patients without thrombocytopenia	Patients with thrombocytopenia	Unadjusted	Adjusted
Bleeding leading to hospitalization				
Any	31.57 (26.80–36.35)	195.53 (145.63–245.42)	1.52 (1.12–2.05)	1.48 (1.09–2.00)
Grade 0	28.72 (23.69– 3.76)	168.57 (115.67–221.48)	1.39 (0.96–2.00)	1.36 (0.94–1.95)
Grade 1	35.87 (19.74–52.00)	240.52 (83.38–397.67)	2.10 (0.92–4.77)	2.06 (0.90–4.71)
Grade 2	62.21 (31.73–92.69)	205.02 (4.10–405.95)	0.95 (0.31–2.90)	0.93 (0.27–3.18)
Grade 3	52.07 (13.50–90.65)	286.02 (0.00–609.69)	1.57 (0.32–7.80)	1.41 (0.24–8.36)
Grade 4	26.53 (0.00–78.54)	1547.7 (30.95–3064.4)	16.23 (1.75–150.44)	13.27 (1.37–128.90)
Grade 5	-	-	-	-
Any transfusion				
Any	18.03 (14.42– 21.64)	308.08 (245.47–370.70)	4.17 (3.11–5.60)	3.91 (2.89–5.30)
Grade 0	14.24 (10.69– 17.78)	180.85 (126.15–235.54)	2.74 (1.83–4.11)	2.50 (1.64–3.79)
Grade 1	26.40 (12.57– 40.23)	319.83 (138.87–500.80)	3.70 (1.70–8.02)	5.03 (2.02–12.50)
Grade 2	50.51 (23.05– 77.97)	673.61 (307.43–1039.8)	4.71 (2.07–10.72)	4.71 (1.91–11.62)
Grade 3	37.19 (4.59– 69.78)	1282.6 (585.38–1979.9)	13.39 (4.30–41.67)	17.02 (4.64–62.48)
Grade 4	53.03 (0.00–126.54)	5261.7 (2284.6–8238.8)	53.18 (6.87–411.72)	57.76 (6.25–534.02)
Grade 5	-	2554.2 (0.00–7560.4)	-	-
Death				
Any	286.31 (271.94–300.68)	974.97 (863.90–1086.0)	5.02 (4.23–5.95)	4.88 (4.11–5.78)
Grade 0	285.11 (269.25–300.96)	769.64 (656.89–882.40)	3.99 (3.25–4.90)	3.89 (3.16–4.78)
Grade 1	280.93 (235.82–326.03)	1087.3 (754.47–1420.1)	6.95 (4.17–11.57)	6.73 (4.03–11.24)
Grade 2	298.87 (232.11–365.63)	1732.5 (1150.1–2314.8)	6.95 (4.01–12.04)	6.96 (3.99–12.13)
Grade 3	297.19 (205.09–389.29)	2545.6 (1585.4–3505.8)	11.78 (5.67–24.46)	12.36 (5.85–26.11)
Grade 4	423.69 (216.08–631.30)	4458.8 (1936.0–6981.6)	17.42 (4.89–62.08)	16.53 (4.52–60.51)
Grade 5	102.48 (0.00–303.35)	7609.4 (0.00– 16220)	12.62 (1.30–122.43)	8.78 (0.78–98.87)

The reference group is a matched comparison cohort without thrombocytopenia. The unadjusted analyses are controlled by matching factors by design. The analyses are adjusted for Charlson Comorbidity Index scores and by matching factors by design.

## Discussion

Our study provided insights into thrombocytopenia occurrence, risk factors, and prognosis among incident solid tumor patients in a population-based setting. The risk of thrombocytopenia was substantial, particularly among patients starting chemotherapy. Risk factors included male sex, advanced stage of cancer, and a high burden of comorbidity. Advanced cancer has been reported to be a risk factor for thrombocytopenia in cancer patients [1]. Old age and many comorbidities such as autoimmune diseases and liver diseases are risk factors for thrombocytopenia [16, 17]. All grades of thrombocytopenia were associated with an increased rate of bleeding leading to hospitalization, transfusion, and death.

Our overall findings are not directly comparable to prior studies, as different definitions of thrombocytopenia were used. As well, earlier research primarily focused on selected patient populations and on chemotherapy-induced thrombocytopenia. For example, one study based on data from the United States Truven Health Analytics MarketScan Database and the IQVIA Real-World Data PharMetrics Database identified all adult patients with primary solid tumors or non-Hodgkin lymphoma who received a course of chemotherapy during 2011-2015 [4]. The incidence of a diagnosis code for thrombocytopenia during the chemotherapy course was 9.7% among all patients in the study population, ranging from 6.1% among patients receiving a cyclophosphamide-based regimen to 13.5% among those receiving a gemcitabine-based regimen [4]. In another study conducted in the United States that examined adult patients with solid tumors treated in outpatient oncology clinics during 2000-2007, the prevalence of thrombocytopenia during the course of chemotherapy ranged from 21.9% in patients treated with taxane-based regimens to 64.2% in patients treated with gemcitabine-based regimens [5]. Our findings on the risk of thrombocytopenia significantly expand the literature, suggesting that the routine clinical care burden of thrombocytopenia among patients with solid tumors and in those receiving chemotherapy is somewhat higher than anticipated.

It is well known that cancer patients are at increased risk of bleeding [18, 19]. The mechanisms are multifactorial, involving anticoagulant therapy, hyperfibrinolysis, tumor invasion, insufficient production of coagulation factors, and presence of other risk factors for bleeding. Although platelet count levels play a pivotal role in bleeding among cancer patients, there is a paucity of data on their prognostic impact. Several studies examined the association between platelet count level and bleeding among patients with hematological cancers [20–25] or in mixed populations encompassing both hematologic malignancies and solid tumors [26–29], but evidence is limited among patients with solid tumors [30–34]. Some research on patients with non-hematological cancers, mostly experimental or in vitro studies, focused on estimating the lowest platelet count resulting in effective hemostasis [35–38]. For example, one study demonstrated that thrombin generation [38] was maintained as long as the platelet count was above 10 × 10^9^/L. At lower levels, thrombin generation declined proportionally to the platelet count. Unfortunately, our data did not allow us to identify the platelet count threshold for effective hemostasis.

Our study size and population coverage, as well as the longitudinal nature and completeness of follow-up characterizing the Danish data sources, are strengths of our study. In addition, the validity of the registry diagnoses that we used in our analyses is high [12]. Laboratory measurements also were performed in accredited hospital laboratories and are thus accurate [11].

Study limitations must be noted. Our data indicated that some clinicians did not measure the platelet count level within the 2 weeks before the record of a cancer diagnosis in the Cancer Registry or, in a few cases, it could indicate that the diagnosis date record was incorrect. Reassuringly, when changing the time window of measurements and when restricting to those who received chemotherapy, our data suggested that the laboratory database is virtually complete. Further, ex vivo agglutination of platelets, while rare, is reported as a text-written result and therefore was not captured by our data sources [11]. A limitation was that a large proportion of patients had missing data on cancer stage. Finally, in the prognosis part of the study, matching was done with replacement, and a substantial number of comparison cohort members were selected several times, leading to CIs that appeared too narrow. Moreover, we did not have data on the severity and specific type of surgical procedures, over the counter medication use, vaccinations, and life style factors. For example, males have been reported to have a lower age-adjusted mean platelet count than females. The reduced platelet count has been suggested to reflect sex difference in hemostasis and effect of smoking on the hemostatic system [39]. Therefore, residual confounding cannot entirely be ruled out.

Our findings may have clinical implications. As there are no drugs for treating thrombocytopenia among patients with cancer, current treatment options are to eliminate/reduce the underlying cause of thrombocytopenia or to provide platelet transfusions. It must be kept in mind that our data were observational and reflected what occurred in routine clinical practice. It is likely that patients with severe thrombocytopenia already received platelet transfusions as prophylactic treatment, which may have blurred and underestimated the true associations between platelet count levels and bleeding. Nonetheless, our data implied that thrombocytopenia is a serious clinical condition in cancer patients.

### Supplementary information

Supplemental Material

## Data Availability

Data presented in this study were obtained from Danish registries. Due to data protection rules, we are not allowed to share individual-level data. Other researchers who fulfill the requirements set by the data providers could obtain similar data.
