# Supplementary material for: Risk and adverse clinical outcomes of thrombocytopenia among patients with solid tumors—a Danish population-based cohort study
Source: Br J Cancer. 2024 Mar 6;130(9):1485–92. doi: 10.1038/s41416-024-02630-w (PMC11058247; doi:10.1038/s41416-024-02630-w)
Supplement: Supplementary file 1 — Supplemental Material [file 41416_2024_2630_MOESM1_ESM.docx]

# Supplementary Material

**Supplementary Table 1**. Description of the data sources used in this study

**Supplementary Table 2.** *International Classification of Diseases, Tenth* Revision (ICD-10) codes used to identify the study population of incident cancer patients

**Supplementary Table 3**. *International Classification of Diseases* (ICD) codes for diseases included in the Charlson Comorbidity Index

**Supplementary Table 4.** NPU codes used in the study

**Supplementary Table 5.** Treatment and comorbidity codes used in the study (Danish National Patient Registry)

**Supplementary Table 6.** Proportion of solid tumor patients with a platelet count measurement, changing the time window for the measurement, by calendar year, Danish region, age group, and cancer type

**Supplementary Table 7.** Cumulative incidence risk of thrombocytopenia, anemia, neutropenia, and leukopenia among solid tumor patients with incident cancer, accounting for the competing risk of death

**Supplementary Table 8.** Number and duration of episodes of thrombocytopenia, anemia, neutropenia, and leukopenia among solid tumor patients with incident cancer and cancer patients initiating chemotherapy. The analysis was restricted to patients with at least one episode of thrombocytopenia, anemia, neutropenia, or leukopenia

**Supplementary Table 9.** Risk factors for thrombocytopenia (<100 x 10^9^/L) among solid tumor patients with incident cancer

**Supplementary Table 10.** Risk factors for thrombocytopenia (<100 x 10^9^/L) among solid tumor patients with incident cancer starting chemotherapy

**Supplementary Table 11.** Adverse clinical outcomes associated with thrombocytopenia among solid tumor patients with incident cancer

**Supplementary Table 12.** Adverse clinical outcomes associated with thrombocytopenia among solid tumor patients with incident cancer starting chemotherapy

**Supplementary Table 13.** All-cause mortality among patients with thrombocytopenia, by combinations of thrombocytopenia, anemia, and neutropenia among solid tumor patients with incident cancer

**Supplementary Table 14.** Adverse clinical outcomes associated with thrombocytopenia among solid tumor patients with incident cancer. Matching 1:1 was performed

**Supplementary Table 15.** Adverse clinical outcomes associated with thrombocytopenia among solid tumor patients with incident cancer initiating chemotherapy. Matching 1:1 was performed.

**Supplementary Figure 1.** Cumulative incidence risk of anemia, neutropenia, and leukopenia among solid tumor patients with incident cancer and among cancer patients initiating chemotherapy

| **Supplementary Table 1**. Description of the data sources used in this study. |
| --- |
| **Danish Civil Registration System:** This registry is updated daily with changes in vital status and migration for the entire Danish population. A unique 10-digit personal identifier is assigned to all residents at birth or upon immigration. This enables accurate and unambiguous linkage across all data sources at the individual level.  **Danish Cancer Registry:** We used the Danish Cancer Registry (DCR) to identify all incident cancers. The DCR has recorded all incident cancer cases in Denmark since 1943, including information on morphology, histology, and stage at diagnosis. Diagnoses are coded according to ICD-10.  **Danish National Patient Registry:** This registry contains complete nationwide information on hospital inpatient admissions since 1977 and on hospital outpatient clinics and emergency contacts since 1995. Each hospital discharge or outpatient visit is recorded with one primary diagnosis and one or more secondary diagnoses coded according to ICD-8 between 1977 and 1993 and according to ICD-10 thereafter.  **Register of Laboratory Results for Research:** Routine biomarker results from the electronic hospital laboratory system covering patients from hospitals and general practitioners are recorded in the register. The database includes variables such as the personal identifier, date and time (hour and minute) of sampling, Nomenclature for Properties and Units (NPU) code, name of the biomarker, identification code for the laboratory and the requisitioner, the test result with the corresponding unit, and the lower and upper reference limits. Most hospital laboratories in Denmark are accredited in accordance with the standards for medical laboratory testing specified by the International Organization for Standardization (ISO 15189). The geographical coverage and start of data collection vary, but from 2015 onwards, the register covers 4 of the 5 Danish regions. |

| **Supplementary Table 2.** *International Classification of Diseases, Tenth* Revision (ICD-10) codes used to identify the study population of incident cancer patients. | |
| --- | --- |
|  | **ICD-10** |
| **All cancers** | C00-96 |
| Mouth and pharynx | C00–C14 |
| Gastrointestinal | C15–C26 |
| Respiratory | C30–C39 |
| Bone | C40–C41 |
| Malignant melanoma | C43 |
| Soft tissue | C45–C49 |
| Breast | C50, D05 |
| Urogenital | C51–C58, C60-C63, C64-68 |
| Central nervous system | C69–C72 |
| Endocrine glands | C73–C75 |
| Unspecified | C76–C80 |
|  |  |
| **Hematological cancers** | C81–C96, D45-D47 |
| Hodgkin lymphoma | C81 |
| Non-Hodgkin lymphomas | C82-C86, C88.0-9, C911b |
| Acute myeloid leukemia | C92 (excl. C921, C922, c927b), C930, C937, C939, C950 |
| Acute lymphoid leukemia | C910 |
| Chronic myeloid leukemia | C921, C922 |
| Chronic lymphocytic leukemia | C911 excl. C911b |
| Multiple myeloma | C90.0 |
| Myeloproliferative neoplasms | D473, D752, D459, D471, D474 |
| Myelodysplastic syndrome | D46, C93 (except C930, C937, C939) |
| Other hematological malignancy | All others codes between C81-C96 not included in the above mentioned classifications |

| **Supplementary Table 3**. *International Classification of Diseases* (ICD) codes for diseases included in the Charlson Comorbidity Index. | | | | |
| --- | --- | --- | --- | --- |
|  | **Disease** | **ICD-8** | **ICD-10** | **Score** |
| 1 | Myocardial infarction | 410 | I21;I22;I23 | 1 |
| 2 | Congestive heart failure | 427.09; 427.10; 427.11; 427.19; 428.99; 782.49 | I50; I11.0; I13.0; I13.2 | 1 |
| 3 | Peripheral vascular disease | 440; 441; 442; 443; 444; 445 | I70; I71; I72; I73; I74; I77 | 1 |
| 4 | Cerebrovascular disease | 430-438 | I60-I69; G45; G46 | 1 |
| 5 | Dementia | 290.09-290.19; 293.09 | F00-F03; F05.1; G30 | 1 |
| 6 | Chronic pulmonary disease | 490-493; 515-518 | J40-J47; J60-J67; J68.4; J70.1;  J70.3; J84.1; J92.0; J96.1; J98.2; J98.3 | 1 |
| 7 | Connective tissue disease | 712; 716; 734; 446; 135.99 | M05; M06; M08; M09;M30;M31;  M32; M33; M34; M35; M36; D86 | 1 |
| 8 | Ulcer disease | 530.91; 530.98; 531-534 | K22.1; K25-K28 | 1 |
| 9 | Mild liver disease | 571; 573.01; 573.04 | B18; K70.0-K70.3; K70.9; K71; K73; K74; K76.0 | 1 |
| 10 | Diabetes type 1    Diabetes type 2 | 249.00; 249.06; 249.07; 249.09  250.00; 250.06; 250.07; 250.09 | E10.0, E10.1; E10.9  E11.0; E11.1; E11.9 | 1 |
| 11 | Hemiplegia | 344 | G81; G82 | 2 |
| 12 | Moderate to severe renal disease | 403; 404; 580-583; 584; 590.09; 593.19; 753.10-753.19; 792 | I12; I13; N00-N05; N07; N11; N14; N17-N19; Q61 | 2 |
| 13 | Diabetes with end organ damage, type1  type2 | 249.01-249.05; 249.08  250.01-250.05; 250.08 | E10.2-E10.8  E11.2-E11.8 | 2 |
| 14 | Any tumor – excluded | 140-194 | C00-C75 | 2 |
| 15 | Leukemia – excluded | 204-207 | C91-C95 | 2 |
| 16 | Lymphoma - excluded | 200-203; 275.59 | C81-C85; C88; C90; C96 | 2 |
| 17 | Moderate to severe liver disease | 070.00; 070.02; 070.04; 070.06; 070.08; 573.00; 456.00-456.09 | B15.0; B16.0; B16.2; B19.0; K70.4; K72; K76.6; I85 | 3 |
| 18 | Metastatic solid tumor – excluded | 195-198; 199 | C76-C80 | 6 |
| 19 | AIDS | 079.83 | B21-B24 | 6 |

| **Supplementary Table 4.** NPU codes used in the study. | |
| --- | --- |
|  | **Laboratory codes** |
| **Platelet count** | NPU03568 (results from automated hematological analyzer) |
| **Leucocytes** | NPU02593 (results from hematological analyzer).  If missing the following code was used (results from microscopy):  NPU17579 |
| **Neutrophils** | Code used in the Capital Region of Denmark and the North Denmark Region  NPU02902 (automated).  If missing the sum of the results for NPU03982+NPU03980 were used (lowest values on the same date). |
| **Hemoglobin** | NPU02319 |

| **Supplementary Table 5.** Treatment and comorbidity codes used in the study (Danish National Patient Registry). | | |
| --- | --- | --- |
|  | **Treatment codes** | **ICD codes** |
| **Transfusion** |  |  |
| Platelet transfusion | BOQC |  |
| Red blood cell transfusion | BOQB |  |
| Plasma transfusion | BOQH |  |
| **Cancer treatment** |  |  |
| Radiation therapy | BWG |  |
| Chemotherapy | BWHA |  |
| Immunotherapy | BWHB, BOHJ |  |
| **Comorbidities** |  |  |
| Bone metastasis |  | C79.5 |
| Liver metastasis |  | C78.7 |
| Liver disease |  | B18, B15.0, B16.0, B16.2, B19.0, I85, K70, K71, K72, K73, K74, K760, K76.6 |
| Disseminated intravascular coagulation |  | D65 |
| Immune thrombocytopenia |  | D693 |
| Hemolytic uremic syndrome/thrombotic thrombocytopenic purpura |  | D593, M311A |
|  |  |  |
| **Bleeding** |  |  |
| Spontaneous and traumatic intracranial bleeding |  | I60-I62, S06.4-S06.6 |
| Bleeding from respiratory tract |  | R04 |
| Gastrointestinal bleeding |  | I85.0, K226, K25.0, K25.2, K25.4, K25.6, K26.0, K26.2, K26.4, K26.6, K27.0, K27.2, K27.4, K27.6, K28.0, K28.2, K28.4, K28.6, K29.0  K62.5, K92.0-K92.2 |
| Bleeding from urinary tract |  | R31.9, N02, |
| Hemorrhagic cystitis |  | N308B |
| Anemia from acute bleeding |  | D62 |

| **Supplementary Table 6.** Proportion of solid tumor patients with a platelet count measurement ever before cancer diagnosis and within two weeks prior to or on their cancer diagnosis. by calendar year, Danish region, age group, and cancer type. | | | | | |
| --- | --- | --- | --- | --- | --- |
|  | **Ever before cancer diagnosis, %** | **Main analysis i.e. from 2 weeks prior to cancer diagnosis to cancer diagnosis, %** | **From 2 weeks prior to cancer diagnosis to 2 weeks after cancer diagnosis, %** | **From 2 weeks prior to cancer diagnosis to any time after cancer diagnosis, %** | **Ever, %** |
| **Calendar year** |  |  |  |  |  |
| 2015 | 76 | 39 | 54 | 91 | 94 |
| 2016 | 87 | 47 | 63 | 92 | 96 |
| 2017 | 90 | 47 | 63 | 89 | 96 |
| 2018 | 94 | 48 | 63 | 80 | 95 |
| **Danish regions** |  |  |  |  |  |
| Capital Region | 93 | 51 | 66 | 90 | 97 |
| Central Region | 92 | 47 | 59 | 86 | 96 |
| Northern Region | 89 | 40 | 59 | 89 | 96 |
| Zealand Region | 83 | 45 | 65 | 90 | 96 |
| Southern Region | 75 | 40 | 54 | 86 | 91 |
| **Age group at cancer diagnosis** |  |  |  |  |  |
| 18 - 30 years | 80 | 29 | 40 | 75 | 87 |
| 31 - 50 years | 81 | 34 | 52 | 82 | 91 |
| 51 - 70 years | 85 | 44 | 60 | 89 | 95 |
| 70+ years | 90 | 51 | 64 | 90 | 97 |
| **Cancer type** |  |  |  |  |  |
| Lip, tongue, oral cavity, salivary gland | 80 | 30 | 37 | 80 | 90 |
| Tongue | 77 | 27 | 49 | 88 | 95 |
| Oral Cavity | 81 | 29 | 49 | 89 | 96 |
| Salivary gland | 80 | 24 | 36 | 87 | 95 |
| Pharyngeal tonsil and cavity of pharynx | 80 | 34 | 72 | 98 | 99 |
| Nasal part of Pharynx | 78 | 38 | 65 | 96 | 100 |
| Other parts of pharynx/ | 83 | 38 | 75 | 98 | 100 |
| Second, poorly specified location in lip, oral cavity and pharynx | 89 | 56 | 67 | 78 | 100 |
| Esophagus | 87 | 53 | 70 | 96 | 99 |
| Stomach | 90 | 61 | 75 | 96 | 99 |
| Small intestine | 93 | 71 | 80 | 98 | 100 |
| Large intestine incl. Colon rectosigmoid | 88 | 54 | 70 | 94 | 97 |
| Rectum | 82 | 42 | 64 | 94 | 97 |
| Anal canal | 83 | 43 | 65 | 97 | 99 |
| Liver | 97 | 79 | 89 | 97 | 99 |
| Gallbladder and biliary tract | 95 | 82 | 89 | 97 | 98 |
| Pancreas | 96 | 81 | 89 | 96 | 99 |
| Second, poorly specified location in digestive organs | 100 | 90 | 93 | 93 | 100 |
| Nasal cavity, middle ear and sinuses | 77 | 28 | 42 | 90 | 94 |
| Larynx | 79 | 30 | 60 | 93 | 97 |
| Lung, bronchi and trachea | 94 | 75 | 91 | 97 | 99 |
| Thymus | 95 | 89 | 98 | 100 | 100 |
| Heart and thoracic cavity | 97 | 72 | 90 | 95 | 95 |
| Pleura | 100 | 75 | 100 | 100 | 100 |
| Second, poorly specified location in airways and respiratory organs | 100 | 100 | 100 | 100 | 100 |
| Bones and articular cartilage | 91 | 48 | 59 | 83 | 95 |
| Malignant melanoma | 85 | 7 | 11 | 54 | 78 |
| Mesothelium, other location | 95 | 77 | 89 | 98 | 99 |
| Kaposis sarcoma | 90 | 75 | 80 | 100 | 100 |
| Peripheral nerves and autonomic nervous system | 88 | 63 | 88 | 100 | 100 |
| Connective tissue | 90 | 51 | 63 | 89 | 95 |
| Breast | 79 | 36 | 68 | 93 | 98 |
| External female genitalia and vagina | 81 | 27 | 42 | 84 | 94 |
| Vagina | 85 | 44 | 67 | 96 | 97 |
| Cervix of uterus | 76 | 28 | 46 | 87 | 94 |
| Uterus | 81 | 26 | 41 | 79 | 92 |
| Ovary | 93 | 70 | 81 | 97 | 99 |
| Placenta | 100 | 33 | 33 | 67 | 67 |
| Second, poorly specified location in female genitalia | 100 | 96 | 96 | 100 | 100 |
| Penis | 83 | 17 | 24 | 74 | 91 |
| Prostate | 85 | 23 | 27 | 77 | 92 |
| Testicle | 76 | 31 | 41 | 78 | 86 |
| Second, poorly specified location in male genitalia | 86 | 21 | 25 | 71 | 82 |
| Kidney | 95 | 65 | 75 | 95 | 99 |
| Renal pelvis and ureter | 91 | 56 | 66 | 91 | 98 |
| Ureter | 91 | 57 | 66 | 97 | 100 |
| Urinary bladder | 90 | 51 | 59 | 92 | 97 |
| Second, poorly specified location in urinary tract | 79 | 50 | 63 | 88 | 100 |
| Eye | 90 | 55 | 67 | 87 | 95 |
| Membrane of the brain and spinal meninx | 93 | 36 | 79 | 79 | 86 |
| Brain | 94 | 78 | 87 | 97 | 99 |
| Spinal cord, cranial nerves and central nervous system | 82 | 50 | 55 | 86 | 95 |
| Thyroid gland | 86 | 27 | 38 | 89 | 97 |
| Adrenal gland | 97 | 62 | 79 | 91 | 100 |
| Other endocrine glands | 88 | 53 | 65 | 88 | 100 |
| Cancer not specified by ICD-10 and other unspecified cancers | 96 | 75 | 83 | 93 | 97 |

| **Supplementary Table 7.** Cumulative incidence risk of thrombocytopenia, anemia, neutropenia, and leukopenia among solid tumor patients with incident cancer, accounting for the competing risk of death. | | | | | | |
| --- | --- | --- | --- | --- | --- | --- |
|  | **Incident cancer cohort** | | | **Cancer patients starting chemotherapy** | | |
|  | Number at risk at 90 days  90-day risk, % (95% CI) | Number at risk at 1 year  1-year risk, % (95% CI) | Number at risk at 4 years  4-year risk, % (95% CI) | Number at risk at 90 days  90-day risk, % (95% CI) | Number at risk at 1 year  1-year risk, % (95% CI) | Number at risk at 4 years  4-year risk, % (95% CI) |
| Thrombocytopenia | 33005  14.46  (14.14-14.77) | 18660  23.27  (22.88-23.66) | 5  29.57  (29.00-30.15) | 19110  33.66  (33.14-34.18) | 10500  42.56  (42.00-43.12) | 0  48.56  (47.81-49.32) |
| Anemia | 13305  51.64  (51.09-52.19) | 6795  64.47  (63.92-65.00) | 5  72.42  (71.64-73.19) | 6765  67.14  (66.50-67.76) | 2945  79.54  (78.98-80.09) | 0  84.48  (83.81-85.12) |
| Neutropenia | 18895  15.22  (14.82-15.62) | 9515  22.91  (22.43-23.38) | 0  27.13  (26.53-27.73) | 10890  53.19  (52.59-53.79) | 4900  60.96  (60.36-61.55) | 0  64.07  (63.40-64.74) |
| Leukopenia | 27415  18.71  (18.35-19.07) | 14590  27.79  (27.37-28.22) | 0  32.67  (32.12-33.22) | 18445  39.90  (39.36-40.44) | 7820  61.02  (60.48-61.57) | 0  70.07  (69.42-70.71) |
| Abbreviation: CI, confidence interval | | | | | | |

| **Supplementary Table 8.** Number and duration of episodes of thrombocytopenia, anemia, neutropenia, and leukopenia among solid tumor patients with incident cancer and cancer patients initiating chemotherapy. The analysis was restricted to patients with at least one episode of thrombocytopenia, anemia, neutropenia, or leukopenia. | | | | |
| --- | --- | --- | --- | --- |
|  | **Median no. of “episodes” during follow-up (25^th^-75^th^ percentiles)** | | **Median duration during follow-up in days (25^th^-75^th^ percentiles)** | |
|  | **Incident cancer cohort** | **Cancer patients initiating chemotherapy** | **Incident cancer cohort** | **Cancer patients initiating chemotherapy** |
| **Thrombocytopenia** |  |  |  |  |
| Any | 1 (1-3) | 2 (1-3) | 16 (8-41) | 16 (9-35) |
| Grade 0 | 1 (1-2) | 1 (1-3) | 16 (8-43) | 16 (9-36) |
| Grade 1 | 1 (1-2) | 1 (1-2) | 14 (8-38) | 14 (8-34) |
| Grade 2 | 1 (1-1) | 1 (1-2) | 12 (7-30) | 13 (8-27) |
| Grade 3 | 1 (1-1) | 1 (1-1) | 11 (7-24) | 12 (8-23) |
| Grade 4 | 1 (1-1) | 1 (1-1) | 12 (7-22) | 13 (8-24) |
| Grade 5 | 1 (1-1) | 1 (1-1) | 12 (7-28) | 14 (8-28) |
| **Anemia (any)** | 1 (1-2) | 1 (1-3) | 73 (28-173) | 88 (39-191) |
| **Neutropenia (any)** | 2 (1-3) | 2 (1-3) | 20 (10-47) | 21 (11-47) |
| **Leukopenia (any)** | 2 (1-3) | 2 (1-3) | 16 (8-41) | 18 (9-44) |

| **Supplementary Table 9.** Risk factors for thrombocytopenia (<100 x 10^9^/L) among solid tumor patients with incident cancer. | | | | | | | |
| --- | --- | --- | --- | --- | --- | --- | --- |
|  | **90-day hazard ratio**  **(95% confidence interval)** | | **1-year hazard ratio**  **(95% confidence interval)** | | **4-year hazard ratio**  **(95% confidence interval)** | |  |
|  | **Unadjusted** | **Adjusted** | **Unadjusted** | **Adjusted** | **Unadjusted** | **Adjusted** |  |
| **Age, years** |  |  |  |  |  |  |  |
| 18-30 | Ref. | Ref. | Ref. | Ref. | Ref. | Ref. |  |
| 31-50 | 0.73 (0.58–0.91) | 0.88 (0.70–1.11) | 0.79 (0.66–0.95) | 0.95 (0.79–1.14) | 0.84 (0.71–1.00) | 1.00 (0.84–1.20) |  |
| 51–70 | 0.96 (0.78–1.18) | 0.93 (0.74–1.16) | 1.01 (0.85–1.20) | 0.99 (0.83–1.19) | 1.08 (0.92–1.27) | 1.07 (0.89–1.27) |  |
| >70 | 0.84 (0.68–1.03) | 0.77 (0.61–0.96) | 0.85 (0.72–1.01) | 0.80 (0.67–0.96) | 0.94 (0.79–1.11) | 0.88 (0.74–1.05) |  |
| **Sex** |  |  |  |  |  |  |  |
| Male | Ref. | Ref. | Ref. | Ref. | Ref. | Ref. |  |
| Female | 0.68 (0.65–0.71) | 0.75 (0.72–0.79) | 0.69 (0.67–0.71) | 0.75 (0.72–0.78) | 0.68 (0.66–0.70) | 0.74 (0.71–0.77) |  |
| **Cancer stage** |  |  |  |  |  |  |  |
| Localized | Ref. | Ref. | Ref. | Ref. | Ref. | Ref. |  |
| Regional | 2.01 (1.88–2.16) | 1.85 (1.72–1.99) | 2.16 (2.04–2.28) | 2.01 (1.89–2.13) | 2.12 (2.01–2.23) | 2.02 (1.92–2.14) |  |
| Metastasis | 2.92 (2.75–3.11) | 2.12 (1.98–2.26) | 3.32 (3.16–3.49) | 2.48 (2.35–2.61) | 3.35 (3.20–3.51) | 2.56 (2.43–2.69) |  |
| Unknown | 1.95 (1.82–2.09) | 1.65 (1.54–1.78) | 1.94 (1.83–2.06) | 1.71 (1.61–1.82) | 1.89 (1.79–1.99) | 1.71 (1.61–1.81) |  |
| Missing | 2.26 (2.04–2.51) | 2.09 (1.69–2.58) | 2.70 (2.49–2.93) | 2.17 (1.80–2.61) | 2.67 (2.47–2.89) | 2.18 (1.82–2.62) |  |
| **Cancer type** |  |  |  |  |  |  |  |
| Mouth and pharynx | 4.44 (3.75–5.27) | 2.51 (1.98–3.18) | 3.33 (2.87–3.86) | 1.82 (1.49–2.23) | 3.13 (2.73–3.59) | 1.73 (1.43–2.09) |  |
| Gastrointestinal | 4.37 (3.92–4.89) | 3.32 (2.96–3.73) | 4.26 (3.91–4.64) | 3.29 (3.01–3.60) | 3.96 (3.67–4.29) | 3.05 (2.81–3.31) |  |
| Respiratory | 5.07 (4.53–5.66) | 3.58 (3.18–4.03) | 4.86 (4.45–5.30) | 3.41 (3.11–3.74) | 4.61 (4.26–4.99) | 3.22 (2.96–3.50) |  |
| Bone | 5.94 (3.82–9.22) | 4.95 (3.18–7.71) | 4.41 (2.96–6.58) | 3.97 (2.66–5.94) | 3.96 (2.71–5.77) | 3.66 (2.51–5.35) |  |
| Malignant melanoma | 1.30 (0.94–1.79) | 1.22 (0.88–1.68) | 1.29 (1.00–1.65) | 1.26 (0.98–1.62) | 1.40 (1.13–1.74) | 1.37 (1.10–1.71) |  |
| Soft tissue | 3.57 (2.92–4.36) | 3.00 (2.45–3.68) | 3.99 (3.42–4.66) | 3.49 (2.98–4.08) | 3.84 (3.33–4.44) | 3.37 (2.91–3.90) |  |
| Breast | Ref. | Ref. | Ref. | Ref. | Ref. | Ref. |  |
| Urogenital | 2.85 (2.53–3.20) | 2.41 (2.13–2.72) | 2.79 (2.55–3.06) | 2.44 (2.21–2.68) | 2.73 (2.51–2.96) | 2.36 (2.16–2.58) |  |
| Central nervous system | 3.77 (3.22–4.43) | 2.40 (1.93–2.98) | 5.39 (4.80–6.05) | 3.39 (2.86–4.03) | 5.28 (4.74–5.88) | 3.43 (2.91–4.03) |  |
| Endocrine glands | 1.20 (0.79–1.81) | 1.01 (0.67–1.54) | 1.33 (0.98–1.80) | 1.17 (0.86–1.58) | 1.27 (0.96–1.66) | 1.12 (0.85–1.48) |  |
| Unspecified | 7.03 (6.09–8.12) | 4.71 (4.05–5.47) | 6.82 (6.05–7.68) | 4.64 (4.10–5.25) | 6.43 (5.75–7.20) | 4.36 (3.88–4.90) |  |
| **CCI score** |  |  |  |  |  |  |  |
| 0 | Ref. | Ref. | Ref. | Ref. | Ref. | Ref. |  |
| 1–2 | 1.10 (1.05–1.16) | 1.01 (0.96–1.06) | 1.06 (1.02–1.10) | 0.99 (0.95–1.03) | 1.08 (1.04–1.12) | 1.01 (0.98–1.05) |  |
| 3+ | 1.57 (1.47–1.68) | 1.32 (1.23–1.41) | 1.42 (1.34–1.50) | 1.26 (1.18–1.33) | 1.44 (1.36–1.52) | 1.28 (1.21–1.35) |  |
| **Cancer treatment*** |  |  |  |  |  |  |  |
| Radiation therapy | 3.24 (2.61–4.04) | 2.64 (2.11–3.30) | 2.59 (2.09–3.22) | 2.06 (1.65–2.57) | 2.47 (2.00–3.06) | 1.93 (1.55–2.39) |  |
| Immunotherapy | 1.43 (1.01–2.01) | 1.27 (0.90–1.80) | 1.35 (1.00–1.83) | 1.20 (0.88–1.63) | 1.34 (1.01–1.79) | 1.20 (0.90–1.60) |  |
| Chemotherapy | 2.01 (1.41–2.87) | 1.98 (1.39–2.82) | 2.21 (1.62–3.02) | 2.15 (1.57–2.94) | 2.13 (1.57–2.90) | 2.09 (1.54–2.85) |  |
| Data are hazard ratios with 95% confidence intervals, adjusted for age, sex, cancer stage, cancer type, Charlson Comorbidity Index score, and cancer treatment.  *Cancer treatment within -30 days before and/+7 days after the index date. For this analysis, the index date was 7 days after the index date to avoid immortal time bias. The comparison was patients without this treatment.  Abbreviation: CCI, Charlson Comorbidity Index | | | | | | |  |

| **Supplementary Table 10.** Risk factors for thrombocytopenia (<100 x 10^9^/L) among solid tumor patients with incident cancer starting chemotherapy. | | | | | | |
| --- | --- | --- | --- | --- | --- | --- |
|  | **90-day hazard ratio**  **(95% confidence interval)** | | **1-year hazard ratio**  **(95% confidence interval)** | | **4-year hazard ratio**  **(95% confidence interval)** | |
|  | **Unadjusted** | **Adjusted** | **Unadjusted** | **Adjusted** | **Unadjusted** | **Adjusted** |
| **Age, years** |  |  |  |  |  |  |
| 18-30 | Ref. | Ref. | Ref. | Ref. | Ref. | Ref. |
| 31-50 | 0.37 (0.32–0.43) | 0.79 (0.67–0.93) | 0.41 (0.35–0.46) | 0.81 (0.70–0.94) | 0.42 (0.37–0.48) | 0.83 (0.71–0.96) |
| 51–70 | 0.56 (0.49–0.64) | 0.88 (0.75–1.03) | 0.62 (0.54–0.70) | 0.89 (0.77–1.04) | 0.65 (0.58–0.74) | 0.92 (0.79–1.06) |
| >70 | 0.65 (0.57–0.74) | 0.92 (0.78–1.09) | 0.72 (0.63–0.82) | 0.93 (0.80–1.08) | 0.76 (0.67–0.86) | 0.95 (0.81–1.10) |
| **Sex** |  |  |  |  |  |  |
| Male | Ref. | Ref. | Ref. | Ref. | Ref. | Ref. |
| Female | 0.63 (0.61–0.66) | 0.83 (0.80–0.87) | 0.63 (0.61–0.65) | 0.82 (0.79–0.86) | 0.62 (0.60–0.64) | 0.82 (0.79–0.86) |
| **Cancer stage** |  |  |  |  |  |  |
| Localized | Ref. | Ref. | Ref. | Ref. | Ref. | Ref. |
| Regional | 1.10 (1.04–1.16) | 1.09 (1.03–1.15) | 1.13 (1.07–1.19) | 1.10 (1.04–1.16) | 1.17 (1.11–1.23) | 1.14 (1.09–1.20) |
| Metastasis | 1.36 (1.29–1.43) | 0.99 (0.94–1.05) | 1.58 (1.51–1.66) | 1.15 (1.09–1.21) | 1.69 (1.62–1.78) | 1.23 (1.17–1.29) |
| Unknown | 1.32 (1.24–1.40) | 1.08 (1.01–1.15) | 1.41 (1.33–1.49) | 1.15 (1.08–1.22) | 1.45 (1.38–1.53) | 1.19 (1.12–1.26) |
| Missing | 1.30 (1.19–1.42) | 1.12 (0.90–1.40) | 1.53 (1.42–1.66) | 1.27 (1.04–1.56) | 1.59 (1.48–1.72) | 1.35 (1.10–1.65) |
| **Cancer type** |  |  |  |  |  |  |
| Mouth and pharynx | 3.84 (3.39–4.35) | 3.18 (2.62–3.86) | 3.27 (2.91–3.67) | 2.59 (2.17–3.10) | 3.10 (2.77–3.47) | 2.45 (2.06–2.92) |
| Gastrointestinal | 3.65 (3.38–3.95) | 3.45 (3.17–3.75) | 3.72 (3.47–3.98) | 3.41 (3.17–3.67) | 3.65 (3.43–3.90) | 3.28 (3.06–3.52) |
| Respiratory | 4.81 (4.44–5.21) | 4.71 (4.32–5.14) | 4.69 (4.38–5.03) | 4.36 (4.04–4.71) | 4.71 (4.41–5.03) | 4.25 (3.95–4.57) |
| Bone | 21.69 (15.53–30.31) | 16.84 (12.00–23.63) | 21.38 (15.40–29.69) | 17.45 (12.52–24.31) | 20.77 (14.97–28.80) | 17.48 (12.57–24.31) |
| Malignant melanoma | 3.30 (2.64–4.11) | 3.04 (2.43–3.80) | 3.41 (2.80–4.14) | 3.14 (2.58–3.82) | 3.34 (2.75–4.05) | 3.07 (2.53–3.73) |
| Soft tissue | 4.08 (3.50–4.74) | 3.73 (3.20–4.36) | 4.33 (3.79–4.94) | 3.93 (3.43–4.49) | 4.48 (3.95–5.08) | 4.03 (3.55–4.59) |
| Breast | Ref. | Ref. | Ref. | Ref. | Ref. | Ref. |
| Urogenital | 4.98 (4.59–5.40) | 4.73 (4.33–5.16) | 4.79 (4.45–5.14) | 4.38 (4.06–4.73) | 4.79 (4.48–5.13) | 4.31 (4.00–4.64) |
| Central nervous system | 3.56 (3.13–4.04) | 2.85 (2.34–3.47) | 4.68 (4.22–5.20) | 3.60 (3.02–4.29) | 4.84 (4.38–5.35) | 3.73 (3.14–4.42) |
| Endocrine glands | 1.69 (1.13–2.52) | 1.46 (0.98–2.17) | 1.58 (1.10–2.27) | 1.35 (0.94–1.95) | 1.51 (1.06–2.15) | 1.29 (0.91–1.83) |
| Unspecified | 5.72 (5.00–6.55) | 5.35 (4.64–6.17) | 5.85 (5.18–6.61) | 5.19 (4.57–5.89) | 5.77 (5.12–6.50) | 4.99 (4.40–5.65) |
| **CCI score** |  |  |  |  |  |  |
| 0 | Ref. | Ref. | Ref. | Ref. | Ref. | Ref. |
| 1–2 | 1.26 (1.21–1.31) | 1.08 (1.04–1.13) | 1.24 (1.20–1.29) | 1.06 (1.02–1.10) | 1.26 (1.21–1.30) | 1.07 (1.03–1.11) |
| 3+ | 1.67 (1.55–1.79) | 1.30 (1.20–1.40) | 1.63 (1.52–1.74) | 1.28 (1.19–1.37) | 1.64 (1.54–1.75) | 1.28 (1.20–1.37) |
| **Cancer treatment*** |  |  |  |  |  |  |
| Radiation therapy | 1.25 (1.19–1.31) | 1.21 (1.15–1.28) | 1.20 (1.15–1.25) | 1.14 (1.08–1.20) | 1.19 (1.14–1.23) | 1.13 (1.07–1.18) |
| Immunotherapy | 0.73 (0.68–0.77) | 0.85 (0.80–0.91) | 0.84 (0.80–0.89) | 0.95 (0.90–1.01) | 0.87 (0.83–0.92) | 0.97 (0.92–1.03) |
| **Specific chemotherapy†** |  |  |  |  |  |  |
| Basic chemotherapy | 4.24 (3.79–4.75) | 1.22 (1.05–1.42) | 4.18 (3.78–4.63) | 1.28 (1.12–1.46) | 3.96 (3.59–4.36) | 1.30 (1.15–1.48) |
| Epirubicine | 1.56 (1.33–1.83) | 0.94 (0.79–1.11) | 1.58 (1.36–1.82) | 0.99 (0.85–1.16) | 1.57 (1.37–1.81) | 1.03 (0.89–1.19) |
| Cisplatine | 5.35 (4.82–5.95) | 1.16 (1.00–1.36) | 5.00 (4.54–5.50) | 1.25 (1.09–1.43) | 4.82 (4.39–5.29) | 1.31 (1.14–1.49) |
| Carboplatin | 6.18 (5.58–6.85) | 1.42 (1.22–1.65) | 6.59 (6.00–7.23) | 1.54 (1.35–1.76) | 6.56 (6.00–7.17) | 1.59 (1.40–1.80) |
| Gemcitabine | 7.31 (6.56–8.15) | 1.04 (0.89–1.22) | 7.88 (7.13–8.70) | 1.14 (0.99–1.32) | 7.93 (7.21–8.72) | 1.20 (1.05–1.38) |
| Cepecitabin | 2.02 (1.80–2.28) | 0.64 (0.54–0.75) | 2.24 (2.02–2.48) | 0.71 (0.61–0.81) | 2.26 (2.05–2.50) | 0.75 (0.66–0.86) |
| Complex chemotherapy | 5.25 (4.67–5.90) | 1.29 (1.11–1.52) | 5.80 (5.22–6.43) | 1.44 (1.25–1.65) | 5.88 (5.33–6.49) | 1.52 (1.33–1.74) |
| Capecitabine+  oxaliplatine | 3.43 (3.05–3.86) | 1.06 (0.91–1.25) | 3.71 (3.34–4.13) | 1.15 (1.00–1.32) | 3.59 (3.25–3.98) | 1.16 (1.02–1.34) |
| carboplatin + vinorelbine | 5.18 (4.66–5.75) | 1.18 (1.01–1.37) | 5.25 (4.77–5.78) | 1.23 (1.08–1.41) | 5.23 (4.77–5.74) | 1.27 (1.11–1.45) |
| Other | 4.06 (3.71–4.43) | 1.07 (0.94–1.23) | 4.44 (4.10–4.81) | 1.20 (1.07–1.36) | 4.48 (4.16–4.83) | 1.26 (1.13–1.41) |
| Data are hazard ratios with 95% confidence intervals, adjusted for age, sex, cancer stage, cancer type, and Charlson Comorbidity Index score.  *Cancer treatment within -30 days before and/+30 days after the index date. For this analysis the index date was 30 days after the index date to avoid immortal time bias  †Compared with cyclophosphamide+epirubicine  Abbreviation: CCI, Charlson Comorbidity Index | | | | | | |

| **Supplementary Table 11.** Adverse clinical outcomes associated with thrombocytopenia among solid tumor patients with incident cancer. | | | | |
| --- | --- | --- | --- | --- |
|  | **Incidence rate per 1000 person-years**  **(95% confidence interval)** | | **Hazard ratio**  **(95% confidence interval)** | |
| **Adverse outcomes by grade** | **Patients without thrombocytopenia** | **Patients with thrombocytopenia** | **Unadjusted** | **Adjusted** |
| **Spontaneous and traumatic intracranial bleeding** |  |  |  |  |
| Any | 7.89 (5.53– 10.25) | 16.61 (0.33– 32.89) | 0.50 (0.18–1.40) | 0.47 (0.17–1.34) |
| Grade 0 | 6.74 (4.33– 9.15) | 15.63 (0.00– 33.33) | 0.54 (0.16–1.79) | 0.52 (0.16–1.73) |
| Grade 1 | 7.34 (0.15– 14.53) | 36.46 (0.00–107.92) | 1.57 (0.16–15.15) | 1.02 (0.09–11.85) |
| Grade 2 | 11.45 (0.00– 24.41) | . (.–.) | . (.–.) | . (.–.) |
| Grade 3 | 42.53 (8.50– 76.57) | . (.–.) | . (.–.) | . (.–.) |
| Grade 4 | . (.–.) | . (.–.) | . (.–.) | . (.–.) |
| Grade 5 | . (.–.) | . (.–.) | . (.–.) | . (.–.) |
| **Respiratory tract bleeding** |  |  |  |  |
| Any | 8.44 (6.00– 10.88) | 54.04 (24.67– 83.42) | 1.42 (0.76–2.65) | 1.41 (0.75–2.64) |
| Grade 0 | 8.31 (5.63– 10.99) | 31.29 (6.25– 56.32) | 0.84 (0.35–2.01) | 0.81 (0.34–1.97) |
| Grade 1 | 9.17 (1.13– 17.21) | 109.45 (0.00–233.31) | 2.81 (0.67–11.80) | 3.50 (0.72–17.13) |
| Grade 2 | 15.27 (0.31– 30.24) | 156.19 (0.00–372.66) | 2.41 (0.44–13.15) | 2.39 (0.33–17.06) |
| Grade 3 | . (.–.) | . (.–.) | . (.–.) | . (.–.) |
| Grade 4 | . (.–.) | . (.–.) | . (.–.) | . (.–.) |
| Grade 5 | - | . (.–.) | . (.–.) | . (.–.) |
| **Gastrointestinal tract bleeding** |  |  |  |  |
| Any | 38.40 (33.19– 43.60) | 345.74 (270.91–420.58) | 2.07 (1.60–2.69) | 1.96 (1.51–2.56) |
| Grade 0 | 38.03 (32.29– 43.76) | 311.68 (232.15–391.22) | 1.86 (1.38–2.52) | 1.80 (1.33–2.44) |
| Grade 1 | 44.09 (26.45– 61.73) | 525.65 (250.30–801.00) | 3.01 (1.54–5.89) | 2.66 (1.30–5.44) |
| Grade 2 | 45.87 (19.91– 71.82) | 314.13 (6.28–621.97) | 1.67 (0.53–5.27) | 1.03 (0.28–3.74) |
| Grade 3 | 14.13 (0.00– 33.72) | 146.69 (0.00–434.19) | 2.50 (0.23–27.57) | 1.65 (0.10–26.87) |
| Grade 4 | 50.39 (0.00–120.23) | 2479.1 (0.00–5284.4) | 7.18 (1.20–43.03) | 10.23 (1.04–100.61) |
| Grade 5 | - | 2075.3 (0.00–6142.8) | (.–.) | (.–.) |
| **Urinary tract bleeding** |  |  |  |  |
| Any | 20.93 (17.09– 24.77) | 133.97 (87.55–180.38) | 1.52 (1.02–2.27) | 1.58 (1.06–2.36) |
| Grade 0 | 20.01 (15.85– 24.17) | 136.64 (84.12–189.16) | 1.58 (1.01–2.45) | 1.62 (1.04–2.53) |
| Grade 1 | 27.55 (13.61– 41.49) | 36.45 (0.00–107.89) | 0.38 (0.05–2.89) | 0.38 (0.05–2.96) |
| Grade 2 | 19.10 (2.36– 35.85) | 236.56 (0.00–504.25) | 3.00 (0.72–12.55) | 2.86 (0.54–15.18) |
| Grade 3 | 28.33 (0.57– 56.10) | 296.95 (0.00–708.50) | 3.00 (0.54–16.60) | 5.22 (0.62–44.04) |
| Grade 4 | 25.20 (0.00– 74.60) | (.–.) | (.–.) | (.–.) |
| Grade 5 | (.–.) | (.–.) | (.–.) | (.–.) |
| **Hemorrhagic cystitis** |  |  |  |  |
| Any | 0.37 (0.00–0.88) | (.–.) | (.–.) | (.–.) |
| Grade 0 | (.–.) | (.–.) | (.–.) | (.–.) |
| Grade 1 | 1.83 (0.00–5.43) | (.–.) | (.–.) | (.–.) |
| Grade 2 | 3.82 (0.00– 11.30) | (.–.) | (.–.) | (.–.) |
| Grade 3 | (.–.) | (.–.) | (.–.) | (.–.) |
| Grade 4 | (.–.) | (.–.) | (.–.) | (.–.) |
| Grade 5 | (.–.) | (.–.) | (.–.) | (.–.) |
| **Anemia from bleeding** |  |  |  |  |
| Any | 1.65 (0.57–2.73) | 24.93 (4.98– 44.87) | 3.69 (1.28–10.64) | 4.10 (1.34–12.53) |
| Grade 0 | 1.57 (0.41–2.74) | 15.64 (0.00– 33.33) | 2.09 (0.54–8.10) | 2.25 (0.53–9.49) |
| Grade 1 | 1.83 (0.00–5.43) | 73.08 (0.00–174.36) | 10.00 (0.91–110.26) | 2.45 (0.15–39.72) |
| Grade 2 | -(.–.) | 77.90 (0.00–230.57) | (.–.) | (.–.) |
| Grade 3 | 7.07 (0.00– 20.92) | (.–.) | (.–.) | (.–.) |
| Grade 4 | (.–.) | (.–.) | (.–.) | (.–.) |
| Grade 5 | (.–.) | (.–.) | (.–.) | (.–.) |
| **Platelet transfusion** |  |  |  |  |
| Any | 1.65 (0.57–2.73) | 120.96 (76.94–164.99) | 15.76 (7.46–33.29) | 15.97 (7.39–34.52) |
| Grade 0 | 1.80 (0.55–3.04) | 52.21 (19.85– 84.57) | 6.02 (2.38–15.28) | 6.02 (2.26–16.08) |
| Grade 1 | (.–.) | 183.80 (22.69–344.91) | (.–.) | (.–.) |
| Grade 2 | 3.82 (0.00– 11.29) | 314.94 (6.30–623.58) | 20.00 (2.24–178.94) | (.–.) |
| Grade 3 | (.–.) | 743.89 (91.84–1395.9) | (.–.) | (.–.) |
| Grade 4 | (.–.) | 3634.3 (72.69–7196.0) | (.–.) | (.–.) |
| Grade 5 | (.–.) | 2006.9 (0.00–5940.3) | (.–.) | (.–.) |
| **Red blood cell transfusion** |  |  |  |  |
| Any | 3.48 (1.92–5.05) | 66.52 (33.93– 99.12) | 4.49 (2.22–9.09) | 4.64 (2.18–9.90) |
| Grade 0 | 3.37 (1.66–5.07) | 26.05 (3.22– 48.89) | 1.44 (0.47–4.41) | 1.64 (0.50–5.40) |
| Grade 1 | 1.83 (0.00–5.43) | 72.89 (0.00–173.91) | 10.00 (0.91–110.26) | (.–.) |
| Grade 2 | 7.63 (0.00– 18.22) | 236.36 (0.00–503.82) | 7.18 (1.20–43.03) | 6.59 (0.46–93.51) |
| Grade 3 | 7.07 (0.00– 20.92) | 588.88 (11.78–1166.0) | (.–.) | (.–.) |
| Grade 4 | (.–.) | 828.23 (0.00–2451.6) | (.–.) | (.–.) |
| Grade 5 | (.–.) | 2052.0 (0.00–6073.8) | (.–.) | (.–.) |
| **Plasma transfusion** |  |  |  |  |
| Any | 2.93 (1.50–4.37) | 95.86 (56.68–135.04) | 6.91 (3.65–13.09) | 8.95 (4.32–18.52) |
| Grade 0 | 2.25 (0.85–3.64) | 83.68 (42.68–124.69) | 7.74 (3.51–17.06) | 8.93 (3.76–21.22) |
| Grade 1 | 3.67 (0.00–8.75) | 109.85 (0.00–234.16) | 7.18 (1.20–43.03) | 8.88 (0.77–102.76) |
| Grade 2 | 15.27 (0.31–30.23) | 155.96 (0.00–372.10) | 2.32 (0.42–12.67) | 4.48 (0.62–32.58) |
| Grade 3 | - | 290.92 (0.00–694.11) | (.–.) | (.–.) |
| Grade 4 | (.–.) | (.–.) | (.–.) | (.–.) |
| Grade 5 | (.–.) | (.–.) | (.–.) | (.–.) |
| The reference group was a matched comparison cohort without thrombocytopenia. The unadjusted analyses were controlled by matching factors by design. The analyses were adjusted for Charlson Comorbidity Index scores and by matching factors by design. | | | | |

| **Supplementary Table 12.** Adverse clinical outcomes associated with thrombocytopenia among solid tumor patients with incident cancer starting chemotherapy. | | | | |
| --- | --- | --- | --- | --- |
|  | **Incidence rate per 1000 person-years**  **(95% confidence intervals)** | | **Hazard ratio**  **(95% confidence intervals)** | |
| **Adverse outcomes by grade** | **Patients without thrombocytopenia** | **Patients with thrombocytopenia** | **Unadjusted** | **Adjusted** |
| **Spontaneous and traumatic intracranial bleeding** |  |  |  |  |
| Any | 1.13 (0.23–2.03) | 3.29 (0.00–9.75) | 0.72 (0.09–5.99) | 1.50 (0.14–16.54) |
| Grade 0 | 1.38 (0.28–2.48) | 4.30 (0.00– 12.73) | 0.72 (0.09–5.99) | 1.50 (0.14–16.54) |
| Grade 1 | (.–.) | (.–.) | . (.–.) | . (.–.) |
| Grade 2 | (.–.) | (.–.) | . (.–.) | . (.–.) |
| Grade 3 | (.–.) | (.–.) | . (.–.) | . (.–.) |
| Grade 4 | (.–.) | (.–.) | . (.–.) | . (.–.) |
| Grade 5 | (.–.) | (.–.) | . (.–.) | . (.–.) |
| **Respiratory tract bleeding** |  |  |  |  |
| Any | 5.45 (3.46–7.43) | 39.59 (17.19– 61.98) | 1.58 (0.77–3.22) | 1.48 (0.71–3.09) |
| Grade 0 | 5.51 (3.31–7.72) | 30.13 (7.81– 52.45) | 1.25 (0.53–2.96) | 1.18 (0.48–2.88) |
| Grade 1 | 5.66 (0.00– 12.06) | 53.16 (0.00–126.83) | 2.28 (0.35–14.95) | 1.30 (0.17–9.75) |
| Grade 2 | 7.76 (0.00– 18.53) | 50.97 (0.00–150.87) | 1.94 (0.17–21.69) | 0.00 (0.00–.) |
| Grade 3 | (.–.) | 94.99 (0.00–281.18) | (.–.) | (.–.) |
| Grade 4 | (.–.) | 378.89 (0.00–1121.5) | (.–.) | (.–.) |
| Grade 5 | (.–.) | (.–.) | (.–.) | (.–.) |
| **Gastrointestinal tract bleeding** |  |  |  |  |
| Any | 12.40 (9.40– 15.39) | 99.09 (63.63–134.55) | 2.13 (1.38–3.30) | 2.03 (1.30–3.16) |
| Grade 0 | 11.94 (8.70– 15.19) | 86.21 (48.43–124.00) | 1.78 (1.06–3.00) | 1.74 (1.03–2.95) |
| Grade 1 | 7.54 (0.15– 14.94) | 186.65 (48.38–324.92) | 7.92 (2.31–27.10) | 10.27 (2.24–47.20) |
| Grade 2 | 27.19 (7.05– 47.34) | (.–.) | (.–.) | (.–.) |
| Grade 3 | 22.30 (0.00– 47.53) | 94.28 (0.00–279.08) | 2.32 (0.21–25.63) | 1.59 (0.09–27.37) |
| Grade 4 | (.–.) | 758.57 (0.00–1809.9) | (.–.) | (.–.) |
| Grade 5 | (.–.) | (.–.) | (.–.) | (.–.) |
| **Urinary tract bleeding** |  |  |  |  |
| Any | 11.83 (8.91– 14.75) | 39.58 (17.18– 61.97) | 0.74 (0.39–1.38) | 0.77 (0.41–1.45) |
| Grade 0 | 9.19 (6.34– 12.03) | 30.13 (0.81– 52.45) | 0.71 (0.31–1.60) | 0.74 (0.32–1.70) |
| Grade 1 | 20.75 (8.49– 33.02) | (.–.) | (.–.) | (.–.) |
| Grade 2 | 27.19 (7.05– 47.32) | 153.72 (0.00–327.68) | 1.56 (0.39–6.28) | 1.41 (0.31–6.29) |
| Grade 3 | 29.74 (0.59– 58.89) | 94.62 (0.00–280.09) | 1.19 (0.13–10.69) | 1.00 (0.08–11.93) |
| Grade 4 | 26.53 (0.00– 78.54) | 371.57 (0.00–1099.8) | 2.24 (0.11–44.88) | 1.80 (0.06–57.98) |
| Grade 5 | (.–.) | (.–.) | . (.–.) | . (.–.) |
| **Hemorrhagic cystitis** |  |  |  |  |
| Any | (.–.) | (.–.) | (.–.) | (.–.) |
| Grade 0 | (.–.) | (.–.) | (.–.) | (.–.) |
| Grade 1 | (.–.) | (.–.) | (.–.) | (.–.) |
| Grade 2 | (.–.) | (.–.) | (.–.) | (.–.) |
| Grade 3 | (.–.) | (.–.) | (.–.) | (.–.) |
| Grade 4 | (.–.) | (.–.) | (.–.) | (.–.) |
| Grade 5 | (.–.) | (.–.) | (.–.) | (.–.) |
| **Anemia from bleeding** |  |  |  |  |
| Any | 1.31 (0.34–2.29) | 19.77 (3.95– 35.59) | 5.76 (1.76–18.91) | 5.65 (1.39–22.92) |
| Grade 0 | 1.38 (0.28–2.48) | 25.81 (5.16– 46.47) | 5.76 (1.76–18.91) | 5.61 (1.40–22.43) |
| Grade 1 | 1.89 (0.00–5.58) | (.–.) | . (.–.) | . (.–.) |
| Grade 2 | (.–.) | (.–.) | (.–.) | (.–.) |
| Grade 3 | (.–.) | (.–.) | (.–.) | (.–.) |
| Grade 4 | (.–.) | (.–.) | (.–.) | (.–.) |
| Grade 5 | (.–.) | (.–.) | (.–.) | (.–.) |
| **Platelet transfusion** |  |  |  |  |
| Any | 3.57 (1.96–5.17) | 132.51 (91.44–173.57) | 9.19 (5.29–15.94) | 10.02 (5.29–19.00) |
| Grade 0 | 2.98 (1.36–4.61) | 43.06 (16.37– 69.75) | 3.39 (1.48–7.76) | 3.77 (1.53–9.31) |
| Grade 1 | 11.31 (2.26– 20.36) | 106.61 (2.13–211.09) | 2.96 (0.83–10.52) | 3.32 (0.72–15.18) |
| Grade 2 | (.–.) | 362.71 (94.01–631.41) | (.–.) | (.–.) |
| Grade 3 | (.–.) | 986.63 (375.11–1598.1) | (.–.) | (.–.) |
| Grade 4 | (.–.) | 3507.8 (1077.0–5938.6) | (.–.) | (.–.) |
| Grade 5 | (.–.) | 2554.2 (0.00–7560.4) | (.–.) | (.–.) |
| **Red blood cell transfusion** |  |  |  |  |
| Any | 15.21 (11.90– 18.53) | 215.94 (163.44–268.44) | 3.61 (2.58–5.05) | 3.43 (2.43–4.84) |
| Grade 0 | 12.17 (8.89– 15.45) | 133.89 (86.76–181.03) | 2.52 (1.59–3.98) | 2.27 (1.41–3.65) |
| Grade 1 | 15.09 (4.63– 25.54) | 241.21 (83.62–398.81) | 5.03 (1.94–13.09) | 7.51 (2.35–23.96) |
| Grade 2 | 50.51 (23.05– 77.97) | 577.35 (236.16–918.54) | 4.06 (1.72–9.59) | 4.07 (1.58–10.45) |
| Grade 3 | 37.19 (4.59– 69.78) | 577.32 (115.37–1039.3) | 5.26 (1.60–17.34) | 8.45 (2.01–35.52) |
| Grade 4 | 53.03 (0.00–126.54) | 3429.6 (1053.0–5806.2) | 36.11 (4.47–291.41) | 46.00 (4.66–453.87) |
| Grade 5 | (.–.) | (.–.) | (.–.) | (.–.) |
| **Plasma transfusion** |  |  |  |  |
| Any | 0.19 (0.00–0.56) | 19.77 (3.95– 35.59) | 28.25 (3.40–234.89) | 23.69 (2.74–204.74) |
| Grade 0 | (.–.) | 8.60 (0.00– 20.52) | (.–.) | (.–.) |
| Grade 1 | 1.89 (0.00–5.58) | (.–.) | (.–.) | (.–.) |
| Grade 2 | (.–.) | 101.95 (0.00–243.25) | (.–.) | (.–.) |
| Grade 3 | (.–.) | 94.33 (0.00–279.22) | (.–.) | (.–.) |
| Grade 4 | (.–.) | 374.23 (0.00–1107.7) | (.–.) | (.–.) |
| Grade 5 | (.–.) | (.–.) | (.–.) | (.–.) |
| The reference group is a matched comparison cohort without thrombocytopenia. The unadjusted analyses were controlled by matching factors by design. The analyses were adjusted for Charlson Comorbidity Index scores and by matching factors by design. | | | | |

| **Supplementary Table 13.** All-cause mortality among patients with thrombocytopenia, by combinations of thrombocytopenia, anemia, and neutropenia among solid tumor patients with incident cancer. | | | | |
| --- | --- | --- | --- | --- |
|  | **Incident cancer cohort** | | **Cancer patients starting chemotherapy** | |
|  | **Hazard ratio**  **(95% confidence interval)** | | **Hazard ratio**  **(95% confidence interval)** | |
|  | **Unadjusted** | **Adjusted** | **Unadjusted** | **Adjusted** |
| Isolated thrombocytopenia (any) | 2.68 (2.29–3.14) | 2.62 (2.24–3.07) | 1.69 (1.17–2.44) | 1.65 (1.14–2.38) |
| Isolated thrombocytopenia (grade 0) | 2.38 (1.99–2.84) | 2.33 (1.95–2.78) | 1.50 (0.98–2.32) | 1.50 (0.97–2.32) |
| Isolated thrombocytopenia (grade 1) | 3.40 (2.21–5.23) | 3.42 (2.21–5.30) | 2.47 (0.93–6.58) | 2.38 (0.88–6.40) |
| Isolated thrombocytopenia (grade 2) | 4.92 (2.35–10.33) | 4.61 (2.17–9.81) | 2.05 (0.68–6.13) | 1.88 (0.55–6.42) |
| Isolated thrombocytopenia (grade 3) | 8.34 (3.07–22.62) | 8.24 (3.03–22.41) | 5.00 (0.31–79.95) | 8.86 (0.22–359.34) |
| Isolated thrombocytopenia (grade 4) | (.–.) | (.–.) | (.–.) | (.–.) |
| Isolated thrombocytopenia (grade 5) | (.–.) | (.–.) | (.–.) | (.–.) |
| Thrombocytopenia with anemia | 4.26 (3.84–4.73) | 4.12 (3.71–4.58) | 5.83 (4.84–7.02) | 5.61 (4.65–6.77) |
| Thrombocytopenia with neutropenia | 2.77 (1.95–3.92) | 2.59 (1.82–3.69) | 4.05 (3.11–5.27) | 3.98 (3.05–5.18) |
| Thrombocytopenia with anemia and neutropenia | 3.36 (2.33–4.83) | 3.26 (2.27–4.70) | 4.75 (3.58–6.30) | 4.56 (3.43–6.06) |
| The unadjusted analyses were controlled by matching factors by design. The analyses were adjusted for Charlson Comorbidity Index scores and by matching factors by design. | | | | |

| **Supplementary Table 14.** Adverse clinical outcomes associated with thrombocytopenia among solid tumor patients with incident cancer. Matching 1:1 was performed. | | | | |
| --- | --- | --- | --- | --- |
|  | **Incidence rate per 1000 person-years**  **(95% confidence interval)** | | **Hazard ratio**  **(95% confidence interval)** | |
| **Adverse outcomes by grade** | **Patients without thrombocytopenia** | **Patients with thrombocytopenia** | **Unadjusted** | **Adjusted** |
| **Bleeding leading to hospitalization** |  |  |  |  |
| Any | 70.53 (54.87– 86.18) | 567.59 (471.13–664.05) | 1.76 (1.31–2.36) | 1.62 (1.19–2.20) |
| Grade 0 | 66.55 (49.71– 83.38) | 507.59 (405.52–609.66) | 1.61 (1.15–2.26) | 1.53 (1.08–2.17) |
| Grade 1 | 80.15 (27.78–132.51) | 759.04 (426.38–1091.7) | 2.25 (0.98–5.17) | 2.96 (0.95–9.25) |
| Grade 2 | 112.79 (22.54–203.04) | 798.71 (303.66–1293.8) | 2.00 (0.68–5.85) | 1.17 (0.29–4.70) |
| Grade 3 | 105.32 (0.00–224.50) | 450.93 (0.00–961.20) | 1.00 (0.20–4.95) | 1.50 (0.25–8.98) |
| Grade 4 | (.–.) | 2479.1 (0.00–5284.4) | (.–.) | (.–.) |
| Grade 5 | (.–.) | 4623.4 (0.00– 11031) | (.–.) | (.–.) |
| **Any transfusion** |  |  |  |  |
| Any | 3.60 (0.07–7.14) | 204.38 (147.15–261.61) | 15.33 (4.77–49.30) | 57.29 (5.22–628.57) |
| Grade 0 | 4.42 (0.09–8.75) | 125.31 (75.18–175.45) | 7.67 (2.30–25.53) | (.–.) |
| Grade 1 | (.–.) | 220.56 (44.08–397.05) | (.–.) | (.–.) |
| Grade 2 | (.–.) | 393.67 (48.60–738.74) | (.–.) | (.–.) |
| Grade 3 | (.–.) | 1190.2 (365.44–2015.0) | (.–.) | (.–.) |
| Grade 4 | (.–.) | 4542.9 (560.87–8524.9) | (.–.) | (.–.) |
| Grade 5 | (.–.) | 2006.9 (0.00–5940.3) | (.–.) | (.–.) |
| **Death** |  |  |  |  |
| Any | 797.34 (744.80–849.87) | 3780.4 (3534.9–4025.9) | 3.43 (2.98–3.96) | 3.43 (2.97–3.96) |
| Grade 0 | 761.52 (704.66–818.38) | 3249.9 (2994.9–3504.9) | 3.04 (2.58–3.59) | 3.03 (2.57–3.57) |
| Grade 1 | 869.28 (697.17–1041.4) | 4690.6 (3881.2–5500.1) | 4.65 (3.05–7.11) | 5.23 (3.23–8.48) |
| Grade 2 | 915.98 (659.50–1172.4) | 6149.8 (4793.6–7505.9) | 4.29 (2.53–7.28) | 4.41 (2.57–7.60) |
| Grade 3 | 1328.7 (906.23–1751.2) | 8406.5 (6243.0– 10570) | 4.73 (2.47–9.06) | 4.68 (2.41–9.10) |
| Grade 4 | 1007.2 (309.26–1705.2) | 13045 (6652.8– 19437) | 7.50 (1.72–32.80) | 15.66 (1.47–167.17) |
| Grade 5 | 1248.0 (0.00–2660.3) | 9979.5 (1232.1– 18727) | (.–.) | (.–.) |
| The reference group is a matched comparison cohort without thrombocytopenia. The unadjusted analyses are controlled by matching factors by design.The analyses are adjusted for Charlson Comorbidity Index scores and by matching factors by design. | | | | |

| **Supplementary Table 15.** Adverse clinical outcomes associated with thrombocytopenia among solid tumor patients with incident cancer initiating chemotherapy. Matching 1:1 was performed. | | | | |
| --- | --- | --- | --- | --- |
|  | **Incidence rate per 1000 person-years**  **(95% confidence interval)** | | **Hazard ratio**  **(95% confidence interval)** | |
| **Adverse outcomes by grade** | **Patients without thrombocytopenia** | **Patients with thrombocytopenia** | **Unadjusted** | **Adjusted** |
| **Bleeding leading to hospitalization** |  |  |  |  |
| Any | 37.61 (26.10– 49.12) | 195.51 (145.62–245.40) | 1.27 (0.84–1.91) | 1.23 (0.80–1.89) |
| Grade 0 | 26.96 (16.17– 37.74) | 168.55 (115.65–221.45) | 1.46 (0.87–2.45) | 1.32 (0.75–2.32) |
| Grade 1 | 91.30 (34.71–147.88) | 240.52 (83.38–397.67) | 0.90 (0.37–2.22) | 1.03 (0.38–2.80) |
| Grade 2 | 93.95 (11.60–176.31) | 205.02 (4.10–405.95) | 0.40 (0.08–2.06) | 0.37 (0.05–2.92) |
| Grade 3 | 72.53 (0.00–173.05) | 286.02 (0.00–609.69) | 1.00 (0.14–7.10) | 1.00 (0.06–15.99) |
| Grade 4 | (.–.) | 1547.7 (30.95–3064.4) | (.–.) | (.–.) |
| Grade 5 | (.–.) | (.–.) | (.–.) | (.–.) |
| **Any transfusion** |  |  |  |  |
| Any | 19.25 (11.02– 27.49) | 308.08 (245.47–370.70) | 3.80 (2.32–6.22) | 3.76 (2.22–6.38) |
| Grade 0 | 16.84 (8.32– 25.37) | 180.85 (126.15–235.54) | 2.36 (1.26–4.40) | 2.17 (1.09–4.29) |
| Grade 1 | 9.11 (0.00– 26.96) | 319.83 (138.87–500.80) | 9.00 (1.14–71.04) | (.–.) |
| Grade 2 | 37.55 (0.00– 89.59) | 673.61 (307.43–1039.8) | 5.50 (1.22–24.81) | 4.00 (0.45–35.79) |
| Grade 3 | 72.38 (0.00–172.69) | 1282.6 (585.38–1979.9) | 5.00 (1.10–22.81) | 4.00 (0.85–18.84) |
| Grade 4 | 130.17 (0.00–385.30) | 5261.7 (2284.6–8238.8) | 12.00 (1.56–92.27) | (.–.) |
| Grade 5 | - | 2554.2 (0.00–7560.4) | (.–.) | (.–.) |
| **Death** |  |  |  |  |
| Any | 368.34 (332.33–404.35) | 974.97 (863.90–1086.0) | 2.84 (2.24–3.60) | 2.76 (2.18–3.50) |
| Grade 0 | 375.96 (335.70–416.22) | 769.64 (656.89–882.40) | 2.24 (1.69–2.95) | 2.16 (1.63–2.86) |
| Grade 1 | 309.68 (205.59–413.78) | 1087.3 (754.47–1420.1) | 4.37 (2.03–9.43) | 4.39 (1.95–9.89) |
| Grade 2 | 431.65 (255.24–608.06) | 1732.5 (1150.1–2314.8) | 3.10 (1.52–6.32) | 3.23 (1.53–6.80) |
| Grade 3 | 288.91 (88.70–489.11) | 2545.6 (1585.4–3505.8) | 11.50 (2.71–48.77) | 15.86 (2.42–104.06) |
| Grade 4 | 260.33 (0.00–621.14) | 4458.8 (1936.0–6981.6) | 12.00 (1.56–92.27) | (.–.) |
| Grade 5 | (.–.) | 7609.4 (0.00– 16220) | (.–.) | (.–.) |
| The reference group is a matched comparison cohort without thrombocytopenia. The unadjusted analyses are controlled by matching factors by design. The analyses are adjusted for Charlson Comorbidity Index scores and by matching factors by design | | | | |

**Supplementary Figure 1.** Cumulative incidence risk of anemia, neutropenia, and leukopenia among solid tumor patients with incident cancer and among cancer patients initiating chemotherapy.

**
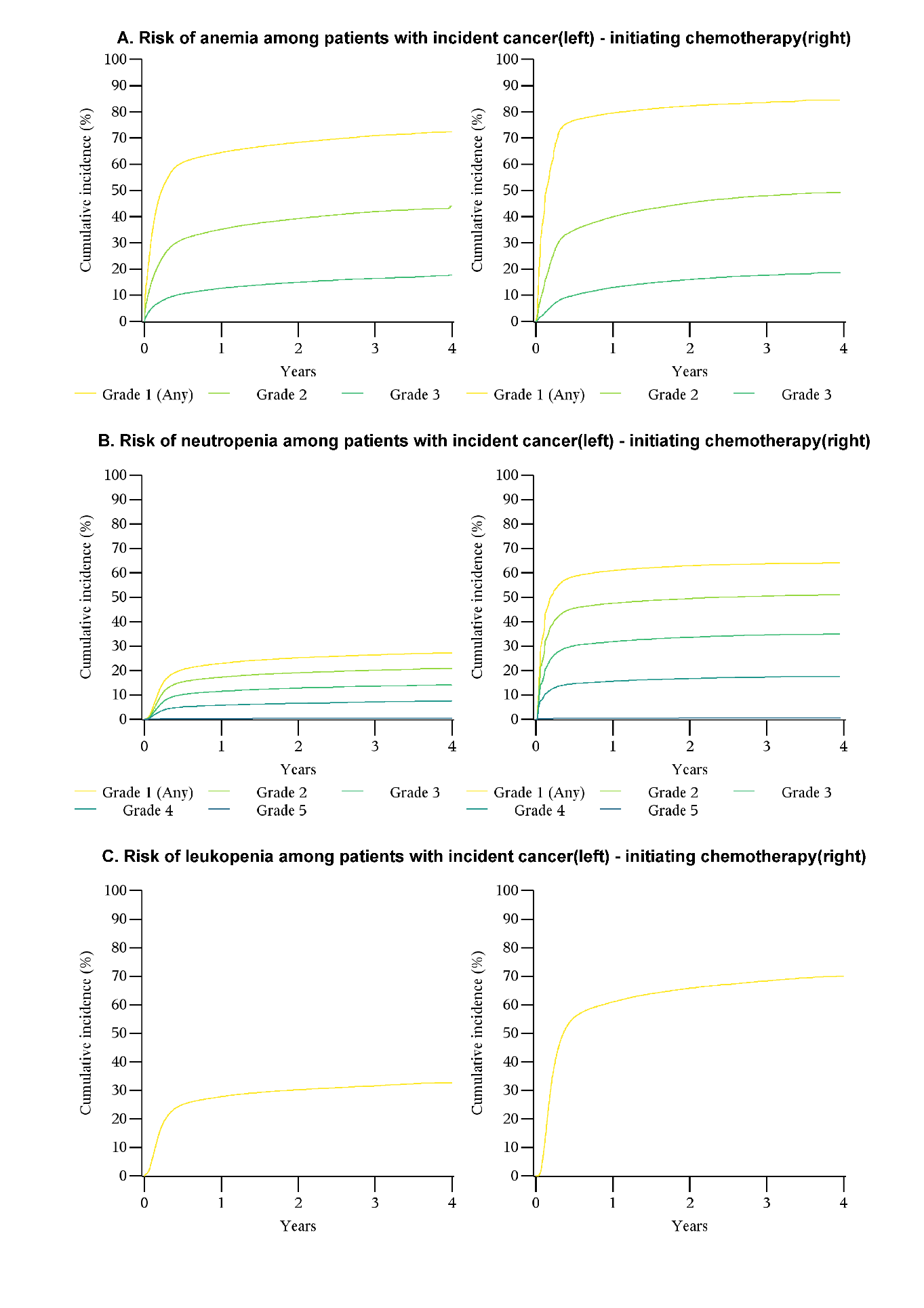
**
